# Engineering *Escherichia coli* for efficient assembly of heme proteins

**DOI:** 10.1186/s12934-023-02067-5

**Published:** 2023-03-28

**Authors:** Jianzhong Ge, Xiaolu Wang, Yingguo Bai, Yaru Wang, Yuan Wang, Tao Tu, Xing Qin, Xiaoyun Su, Huiying Luo, Bin Yao, Huoqing Huang, Jie Zhang

**Affiliations:** State Key Laboratory of Animal Nutrition, Institute of Animal Science, Chinese Academy of Agricultural Sciences, No.2 Yuanmingyuan West Road, Haidian District, Beijing, 100193 China

**Keywords:** Heme, Dye-decolorizing peroxidase, Oxygen-transport protein, Cytochrome P450, Whole-cell bioconversion

## Abstract

**Background:**

Heme proteins, such as hemoglobin, horseradish peroxidase and cytochrome P450 (CYP) enzyme, are highly versatile and have widespread applications in the fields of food, healthcare, medical and biological analysis. As a cofactor, heme availability plays a pivotal role in proper folding and function of heme proteins. However, the functional production of heme proteins is usually challenging mainly due to the insufficient supply of intracellular heme.

**Results:**

Here, a versatile high-heme-producing *Escherichia coli* chassis was constructed for the efficient production of various high-value heme proteins. Initially, a heme-producing *Komagataella phaffii* strain was developed by reinforcing the C4 pathway-based heme synthetic route. Nevertheless, the analytical results revealed that most of the red compounds generated by the engineered *K. phaffii* strain were intermediates of heme synthesis which were unable to activate heme proteins. Subsequently, *E. coli* strain was selected as the host to develop heme-producing chassis. To fine-tune the C5 pathway-based heme synthetic route in *E. coli*, fifty-two recombinant strains harboring different combinations of heme synthesis genes were constructed. A high-heme-producing mutant *Ec*-M13 was obtained with negligible accumulation of intermediates. Then, the functional expression of three types of heme proteins including one dye-decolorizing peroxidase (Dyp), six oxygen-transport proteins (hemoglobin, myoglobin and leghemoglobin) and three CYP153A subfamily CYP enzymes was evaluated in *Ec*-M13. As expected, the assembly efficiencies of heme-bound Dyp and oxygen-transport proteins expressed in *Ec*-M13 were increased by 42.3–107.0% compared to those expressed in wild-type strain. The activities of Dyp and CYP enzymes were also significantly improved when expressed in *Ec*-M13. Finally, the whole-cell biocatalysts harboring three CYP enzymes were employed for nonanedioic acid production. High supply of intracellular heme could enhance the nonanedioic acid production by 1.8- to 6.5-fold.

**Conclusion:**

High intracellular heme production was achieved in engineered *E. coli* without significant accumulation of heme synthesis intermediates. Functional expression of Dyp, hemoglobin, myoglobin, leghemoglobin and CYP enzymes was confirmed. Enhanced assembly efficiencies and activities of these heme proteins were observed. This work provides valuable guidance for constructing high-heme-producing cell factories. The developed mutant *Ec*-M13 could be employed as a versatile platform for the functional production of difficult-to-express heme proteins.

**Supplementary Information:**

The online version contains supplementary material available at 10.1186/s12934-023-02067-5.

## Background

Heme is composed of four porphyrin ligands chelated with a ferrous ion [1]. As a cofactor for various functional proteins, heme is essential for nearly all physiological processes of cellular life, such as blood cell differentiation [2], lipid metabolism [3], iron metabolism [4] and energy generation [5]. Heme synthesis pathway exists in most organisms except for a few species that rely on the acquisition and utilization of exogenous heme, such as lactic acid bacteria [6], ticks [7], or *Caenorhabditis elegans* [8]. Heme is mostly known as the oxygen binding prosthetic group in hemoglobin for delivering oxygen. It has been reported that the application of acellular hemoglobin in artificial blood can effectively alleviate blood deficiency and eliminate infectious diseases caused by the delivery of natural blood [9]. In addition, *Vitreoscilla* hemoglobin can be used in microbial high-cell-density fermentation because of its high oxygen delivery capacity [10]. In recent years, heme proteins have shown great potential to solve diverse problems associated with food crisis and safety. Currently, the soybean hemoglobin, which could provide high quality protein with less carbon footprint and make artificial meat more authentic in color and flavor, has been approved by the FDA as a food additive [11]. Mycotoxins secreted by filamentous fungi in cereals have been reported to cause serious harm to human and animal health [12]. Several heme proteins, such as manganese peroxidases and dye-decolorization peroxidases, could metabolize a wide range of substrates and provide good solutions for degrading mycotoxins in grain in a green manner [13, 14].

**Fig. 1:**
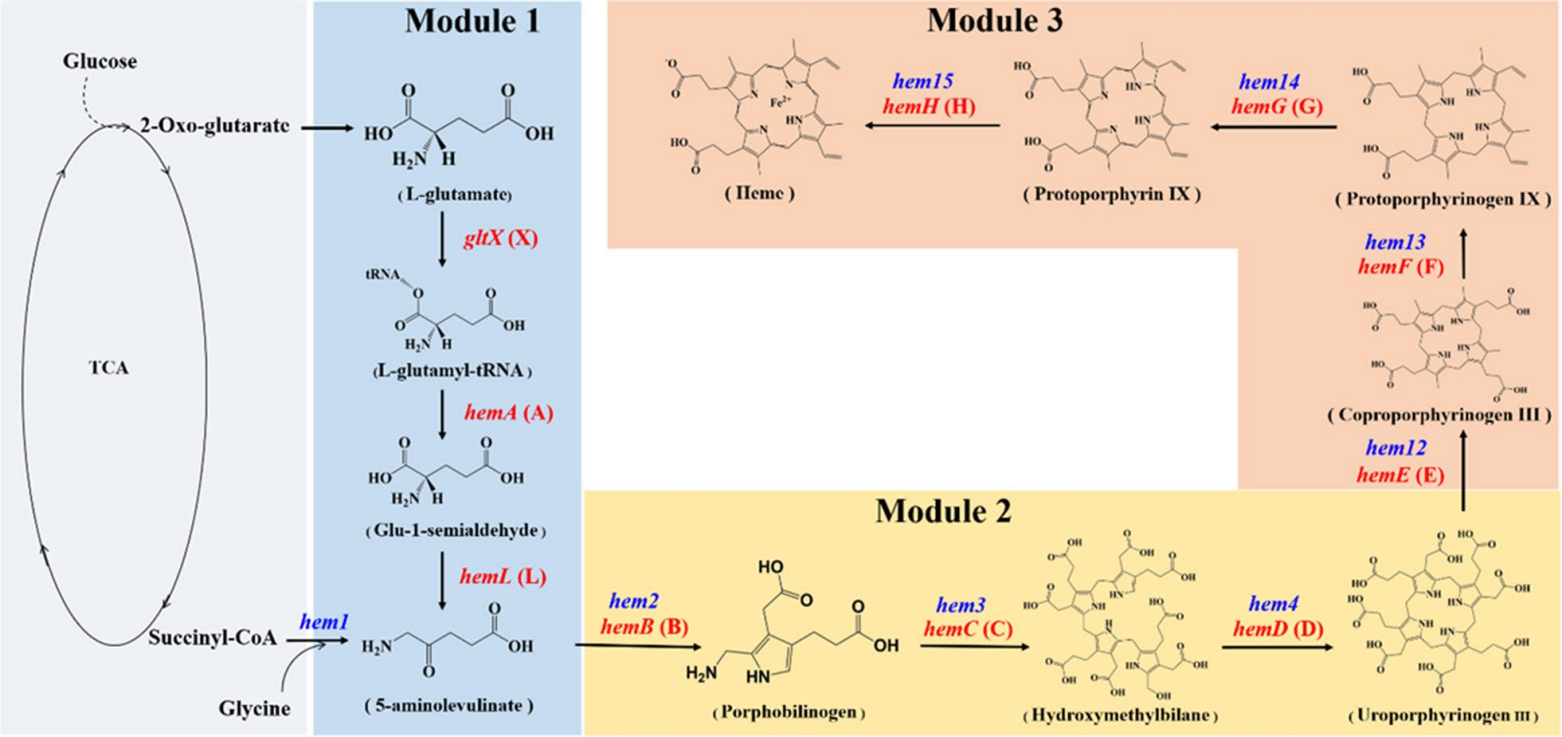
Heme biosynthetic pathways in *K. phaffii* and *E. coli*. The pathways emphasized in gray, blue, yellow and orange indicate the modules of tricarboxylic acid (TCA) cycle, 5-aminolevulinate (Module 1), tetrapyrrole (Module 2) and downstream heme biosynthesis pathway (Module 3), respectively. The corresponding metabolic genes indicated in blue and red were overexpressed in *K. phaffii* and *E. coli* recombinants, respectively, for promoting heme synthesis. *gltX*, glutamyl-tRNA synthetase gene; *hemA*, glutamyl-tRNA reductase gene; *hemL*, glutamate-1-semialdehyde-2,1-aminomutase gene; *hem1*, 5-aminolevulinate synthase gene; *hemB* (*hem2*), delta-aminolevulinic acid dehydratase gene; *hemC* (*hem3*), porphobilinogen deaminase gene; *hemD* (*hem4*), uroporphyrinogen-III synthase gene; *hemE* (*hem12*), uroporphyrinogen decarboxylase gene; *hemF* (*hem13*), coproporphyrinogen-III oxidase gene; *hemG* (*hem14*), protoporphyrinogen IX dehydrogenase gene; *hemH* (*hem15*), ferrochelatase gene

The heme synthesis pathway is complex and varies in different organisms [15]. Using the synthesis routes of heme in *Komagataella phaffii* and *Escherichia coli* as an example, briefly, 5-aminolevulinate (5-ALA) derived from intermediates of tricarboxylic acid (TCA) cycle via C4 or C5 pathway is used as the substrate to form uroporphyrinogen III under the catalysis of delta-aminolevulinic acid dehydratase (HEM2/HemB), porphobilinogen deaminase (HEM3/HemC) and uroporphyrinogen-III synthase (HEM4/HemD) (Fig. 1). This process is highly conserved and is known as the tetrapyrrole synthesis pathway. However, the synthesis pathway of uroporphyrinogen III to heme is significantly different among species. According to the signature intermediates, the pathways from urinary uroporphyrinogen III to heme can be classified into three types, including siroheme-, coproporphyrin- and protoporphyrin-dependent pathways. Among them, the protoporphyrin-dependent branch which was long believed to be the sole biosynthetic pathway for heme is considered as the classic pathway. In this pathway, uroporphyrinogen III is converted into protoporphyrin IX through decarboxylation and oxidation catalyzed by uroporphyrinogen decarboxylase (HEM12/HemE), coproporphyrinogen-III oxidase (HEM13/HemF), and protoporphyrinogen IX dehydrogenase (HEM14/HemG). Finally, protoporphyrin IX chelates a ferrous ion to form ferrous heme under the catalysis of ferrochelatase (HEM15/HemH). In addition, uroporphyrinogen III can also be used to generate coenzyme F430, cobalamin and chlorophyll after chelating with nickel, cobalt and magnesium atom, respectively.

Efficient synthesis of heme is one of the major challenges for the production of heme proteins. Tremendous efforts have been made to construct the efficient microbial cell factory for heme and heme proteins production. Overexpression of 5-ALA synthase from *Rhodobacter sphaeroides* which is the key enzyme in C4 pathway could enhance the heme production in *E. coli* [16, 17]. By optimizing the expression level of heme synthesis related genes, knocking down a heme degradation enzyme and overexpressing heme export proteins, the heme production in *E. coli* reached 239.2 mg/L [18]. Recently, up to 84 genes in *Saccharomyces cerevisiae* which were involved in heme synthesis, glycolysis pathway and metabolism of pyruvate, Fe-S clusters, glycine, and succinyl-CoA were highlighted by in silico simulation using a genome-scale metabolic model [19]. Among them, 40 genes were found to have positive effects on heme biosynthesis and the heme production of the optimized strain increased by about 70-fold. For heme proteins production, the most extensively studied heme protein is hemoglobin [20]. To meet the increasing demand for hemoglobin, microbial synthesis platforms have recently become more attractive due to higher titers, yields, and productivities. The microbial species used to express hemoglobin mainly include *E. coli*, *S. cerevisiae* and *K. phaffii* [20]. However, the insufficient supply of heme, production of inclusion bodies and easy degradation pose huge challenges to heme proteins production. In the case of hemoglobin, more than 100,000 nucleotide sequences have been discovered, however very few of them have been successfully expressed and characterized, including those from human and soybean [20]. To address these challenges, various strategies have been applied, including exogenous supply of heme, co-expression of molecular chaperones, optimization of codons and fermentation conditions [21].

In this study, we aimed to construct a robust heme-producing microbial chassis for various heme proteins production. Initially, the heme synthesis pathway was enhanced in *K. phaffii*. However, a large amount of heme synthesis intermediates, instead of heme itself, were found to be accumulated in the engineered *K. phaffii* mutant and these intermediates could not activate heme proteins. A similar phenomenon was also observed in *E. coli*. In order to reduce the accumulation of heme synthesis intermediates, the effects of overexpression of different combinations of heme synthesis genes on intermediates and heme productions were investigated. Finally, the functional expression of different types of heme proteins including *Bacillus subtilis* dye-decolorizing peroxidase (*Bs*Dyp), oxygen-transport proteins and cytochrome P450 (CYP) enzymes were evaluated in the obtained high-heme-producing *E. coli* strain.

## Results and discussion

### Attempts of construction of heme- and heme protein-producing *K. phaffii* strains

*K. phaffii* has been widely employed as a powerful platform for the expression of thousands of recombinant proteins, including various heme proteins [22]. In order to achieve high production of heme proteins, the heme supply should be sufficient. *K. phaffii* harbors a native synthetic pathway for heme production. However, the biosynthesis of heme in *K. phaffii* is tightly regulated, resulting in extremely low level of intracellular heme which would be difficult to meet the requirement for high production of heme proteins [22]. Therefore, firstly we sought to construct a high heme-producing *K. phaffii* strain. The C4 pathway-based heme synthetic route in *K. phaffii* GS115 was reinforced by overexpressing 8 endogenous genes including *hem1*-*hem4* and *hem12*-*hem15* (Fig. 1), generating mutants *Pp*H4 (harboring *hem1*-*hem4*) and *Pp*H8 (harboring *hem1*-*hem4* and *hem12*-*hem15*).

**Fig. 2:**
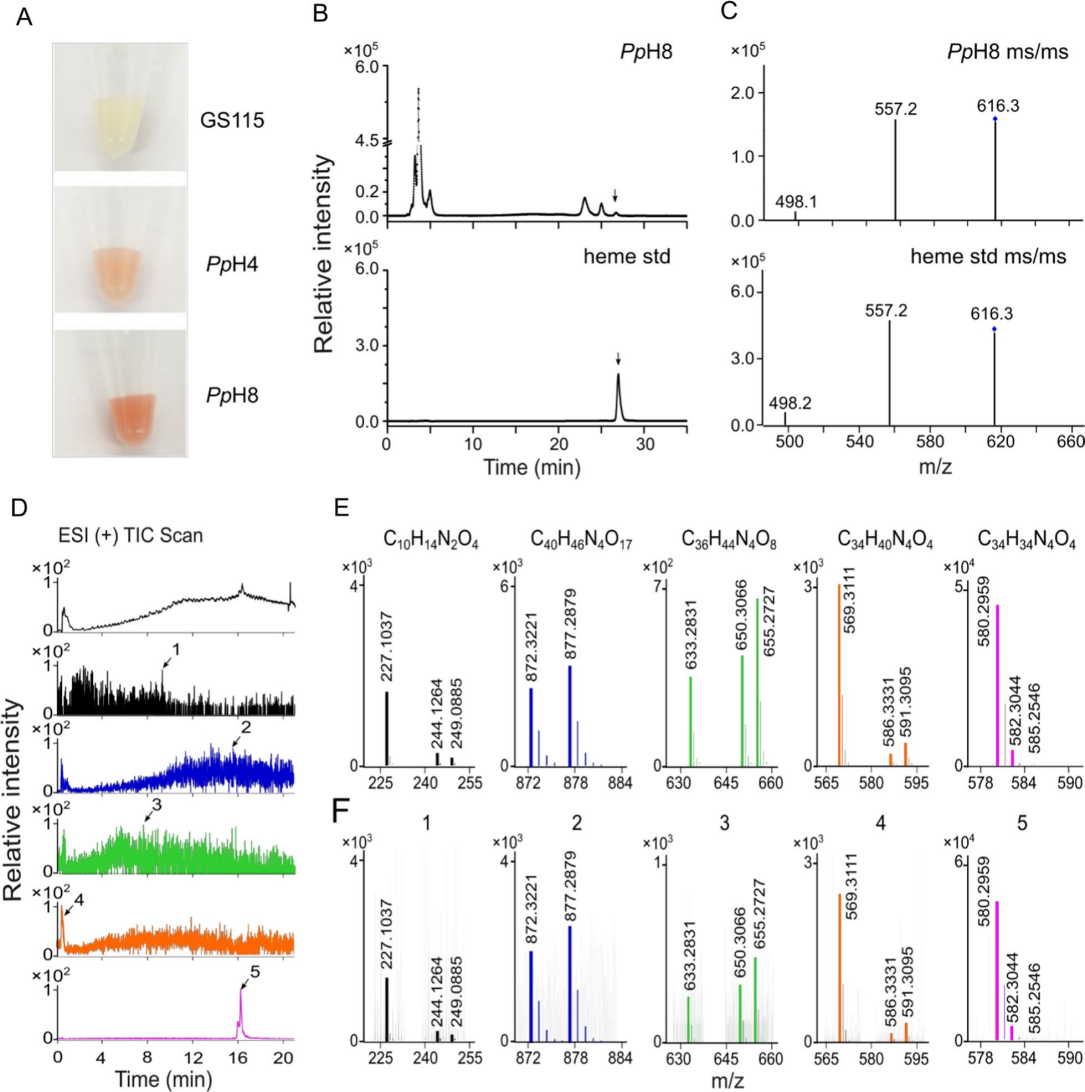
Characterization of fermentation products of heme-producing *K. phaffii* strains. **A** The fermentation cultures of GS115, *pP*H4 and *pP*H8 strains. **B** HPLC analysis of fermentation extract of *pP*H8 strain and heme standard (heme std) at 400 nm. **C** ESI–MS/MS chromatogram analysis of heme produced by *pP*H8 (indicated by the arrow in **B** and heme std. **D** ESI ( +) EIC analysis of fermentation products of *pP*H8 strain. **E** ESI–MS/MS chromatogram analysis of chemical standards including porphobilinogen (C_10_H_14_N_2_O_4_), hydroxymethylbilane (C_40_H_46_N_4_O_17_), coproporphyrinogen III (C_36_H_44_N_4_O_8_), protoporphyrinogen IX (C_34_H_40_N_4_O_4_) and protoporphyrin IX (C_34_H_34_N_4_O_4_). **F** ESI–MS/MS chromatogram analysis of fermentation product of *pP*H8 strain indicated by the corresponding arrows in **D**

The obtained strains were then characterized in BMMY medium. Results showed that the fermentation cultures of *Pp*H4 and *Pp*H8 exhibited red color (Fig. 2A), indicating the formation of heme-like products. It is reported that heme-like product has a characteristic Soret peak at 400 nm [18]. Therefore, the fermentation product of *Pp*H8 was further analyzed using UV–visible spectroscopy. Similar as the heme standard, an obvious absorbance peak at 400 nm was observed for the product of *Pp*H8 (Additional file 1: Fig. S1). In order to further increase the heme production in *Pp*H8 strain, the effects of supplementation of different precursors (5-ALA, glycine and iron ions, 0.5–2.0 mM) and carbon sources (glucose, glycerol and methanol, 2–4%) on heme synthesis were investigated. Compared with the control, adding 1.0 mM 5-ALA, 1.0–2.0 mM Fe^2+^, 0.5–2.0 mM glycine, 2–4% glucose, 3% glycerol or 2–4% methanol in the medium slightly increased the 400 nm absorbance of fermentation products of *Pp*H8 (Additional file 1: Figs. S2 and S3). These results suggested that precursors supplementation and carbon sources optimization could have positive effects on the synthesis of heme-like products.

**Fig. 3:**
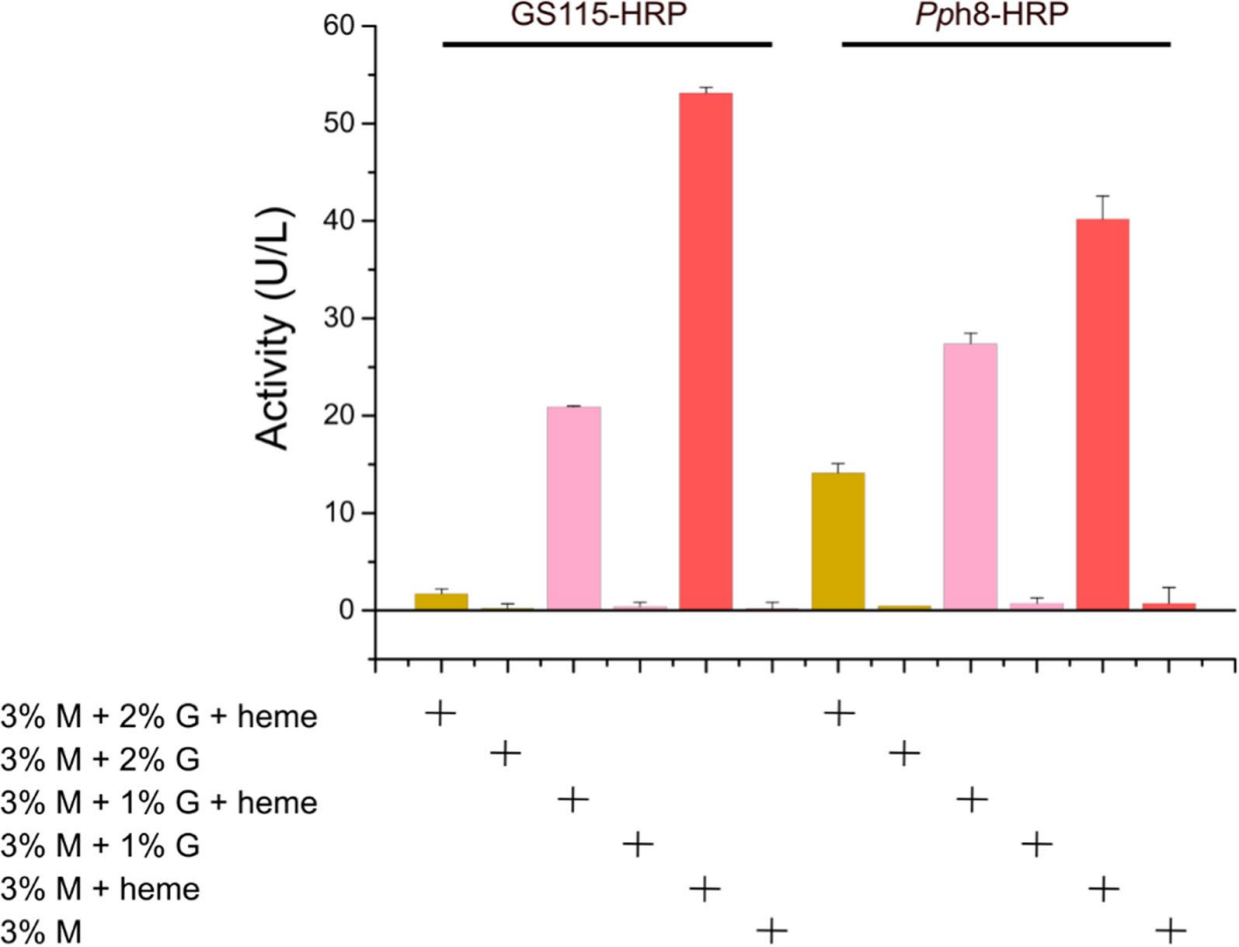
The horseradish peroxidase (HRP) activities of GS115-HRP and *pP*H8-HRP cultured in BMMY medium containing different combinations of methanol (M), glucose (G) and exogenous heme (20 μM)

Horseradish peroxidase (HRP) is one of the most important heme-containing enzymes that can utilize H_2_O_2_ to oxidize various organic and inorganic compounds and has been widely used in the fields of immunoassays and biosensors [23]. Therefore, HRP was selected as the target protein to be overexpressed in *Pp*H8 to verify whether heme-producing strain is beneficial for the production of heme proteins. The *hrp* gene was expressed under the control of strong methanol inducible promoter (AOX1) and integrated into the genomes of GS115 and *Pp*H8 strains, yielding GS115-HRP and *Pp*H8-HRP mutants, respectively. Shake-flask fermentations with GS115-HRP and *Pp*H8-HRP were then carried out in BMMY medium containing different combinations of methanol, glucose and heme (Fig. 3). The results showed that the HRP activities of both GS115-HRP and *Pp*H8-HRP were extremely low when no exogenous heme was supplemented in the medium. In contrast, with the addition of exogenous heme, the HRP activities of GS115-HRP and *Pp*H8-HRP were significantly increased, suggesting that the heme production in *Pp*H8 strain was not satisfied with the requirement for functional production of HRP enzyme. In addition, the presence of glucose in the fermentation medium severely inhibited the expression of *hrp* gene driven by the methanol inducible AOX1 promoter (Fig. 3). When methanol was used as the sole carbon source and the exogenous heme was added in the medium, the HRP activities of GS115-HRP and *Pp*H8-HRP reached their maximum values of 53.1 and 40.2 U/L, respectively, indicating that *Pp*H8 was not a suitable host for heme protein production compared with the wild-type strain.

It is known that heme contains four porphyrin ligands and some of the heme-like intermediates also exhibit red color and have characteristic peak at 400 nm. Therefore, the fermentation products of *Pp*H8 were further analyzed by HPLC-MS to verify the structures of the substances which displayed an absorption peak at 400 nm. The results showed that the heme absorption peak area only accounted for about 1% of total peak area at 400 nm (Fig. 2B, C). Interestingly, a large amount of intermediate metabolites in the heme biosynthesis, including protoporphyrin IX and protoporphyrinogen IX, were found (Fig. 2D, E, F). Collectively, overexpression of the genes involved in heme synthesis pathway in *K. phaffii* could enhance the formation of heme-like products (showed red color and had absorption peak at 400 nm), but heme synthesis intermediates, instead of heme itself, were prone to be accumulated mainly due to the cumbersomeness of the pathway. Therefore, the expression of heme synthesis genes should be optimized to minimize the accumulation of intermediate products and the possible metabolic burden to the host.

### Construction of heme-producing *E. coli* strains

As an efficient microbial cell factory and the most characterized gram-negative bacterium, *E. coli* has also been extensively used for the production of various heme proteins [20]. Compared to *K. phaffii* strain, *E. coli* has much simpler cell structure and more mature genetic manipulation methods. Therefore, *E. coli* was selected as another host to construct high heme-producing recombinant. Different from *K. phaffii*, the 5-ALA synthetic route in *E. coli* is depended on C5 pathway and thus 10 genes are involved in the heme synthetic pathway of *E. coli* (Fig. 1), including *gltX* (X), *hemA* (A), *hemL* (L), *hemB* (B), *hemC* (C), *hemD* (D), *hemE* (E), *hemF* (F), *hemG* (G) and *hemH* (H). Initially, the 10 genes were overexpressed individually to investigate their role in heme biosynthesis. However, overexpression of any one of these genes alone could not significantly increase the heme production (Additional file 1: Fig. S4). To evaluate the effects of overexpression of different combinations of the 10 genes on heme biosynthesis, the heme synthesis pathway was divided into three modules based on the important intermediates (Fig. 1) [15]. Module 1 contained X, A and L genes; Module 2 contained B, C and D genes; Module 3 contained E, F, G and H genes. The genes in each module were also overexpressed in different combinations. Therefore, total 29 overexpression plasmids were constructed and tested in *E. coli* BL21(DE3), generating 52 recombinant strains (Additional file 1: Tables S1 and S2).

**Fig. 4:**
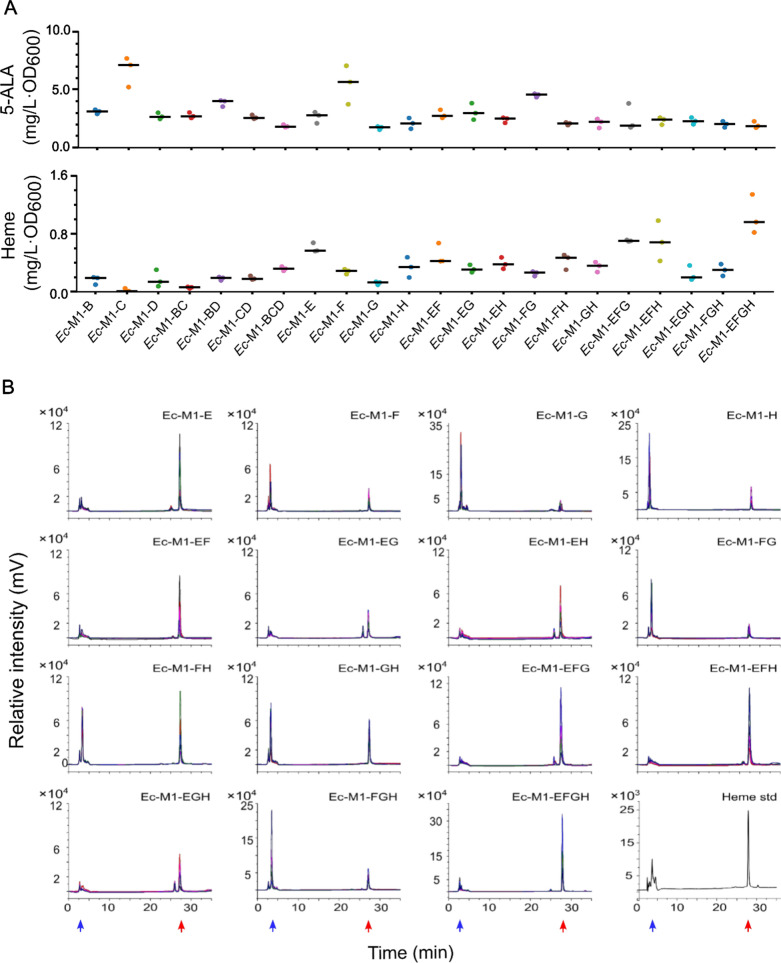
Heme and intermediates productions of *E. coli* recombinants overexpressing different combinations of heme synthesis genes. **A** 5-ALA and heme productions. **B** HPLC analysis of fermentation extracts at 400 nm. The heme and intermediates were marked with red and blue arrows, respectively. Five independent HPLC profiles were provided for each recombinant. M1 contains *gltX*, *hemA* and *hemL* genes; B, *hemB*; C, *hemC*; D, *hemD*; E, *hemE*; F, *hemF*; G, *hemG*; H, *hemH*; *Ec*-M1-BCD, *Ec*-M12; *Ec*-M1-EFGH, *Ec*-M13; 5-ALA, 5-aminolevulinate

Fermentation results demonstrated that overexpression of genes involved in any single module had negligible effect on heme production (Additional file 1: Fig. S4). Therefore, fermentations with recombinant strain *Ec*-M123 harboring three expression plasmids and containing all 10 genes were carried out in the LBFG medium supplemented with different concentrations of three antibiotics to regulate the overexpression levels of the genes in the three modules (Fig. 1 and Additional file 1: Fig. S6). The results showed that the expression level of module 1 genes was positively correlated with the heme production. The highest heme titer reached 0.55 mg/L·OD_600_, which was increased by 1.75-fold compared to that obtained at low antibiotic condition. Overexpression of module 1 genes could enhance the synthesis of 5-ALA (Fig. 4A), the precursor of heme, suggesting that increasing 5-ALA production was essential for promoting heme synthesis [24]. However, similar with the results obtained by *K. phaffii* strain, small amount of intermediate metabolites were generated during the fermentation of *Ec*-M123 (Additional file 1: Fig. S6), indicating that the expression of the 10 genes occurred in an uncoordinated fashion.

To reduce the production of intermediate metabolites and the metabolic burden to the host strain, the genes in module 1 were co-expressed with the genes in module 2 or module 3 to confirm the necessity of overexpressing the 7 genes involved in module 2 and module 3. Fermentation results showed that the heme production of *Ec*-M1-EFGH (*Ec*-M13) strain was 2.27-fold higher than that of *Ec*-M1-BCD (*Ec*-M12) strain (Fig. 4A). In addition, the heme accumulation was more significantly upregulated by module 3 genes than module 2 genes (Fig. 4A, B), indicating that there might be rate-limiting steps for heme synthesis in module 3. The heme titers of *Ec*-M1-E, *Ec*-M1-EFG, *Ec*-M1-EFH and *Ec*-M1-EFGH reached 0.60, 0.70, 0.70 and 1.04 mg/L·OD_600_, respectively, which were enhanced by 9.1%, 27.3%, 27.3% and 89.1%, respectively, compared to that of *Ec*-M123 strain harboring all 10 genes involved in module 1, module 2 and module 3. It is worthwhile to point out that co-expression of module 1 genes with only *hemE* gene (strain *Ec*-M1-E) could achieve a better performance on heme production than overexpression of all 10 genes (strain *Ec*-M123), and no significant accumulation of intermediates was detected in the fermentation of *Ec*-M1-E (Fig. 4B), indicating that *hemE* gene in module 3 played a key role in the regulation of heme synthesis. Among the four high-heme-producing strains, *Ec*-M1-EFGH (*Ec*-M13) produced the highest amount of heme and the lowest amount of intermediates (Fig. 4A and B). Therefore, this strain could be employed as a suitable and robust host for efficient production of heme proteins. It is reported that expression of heme exporter could enhance heme efflux and further improve heme production [18]. However, the aim of this study was to construct a platform for intracellular heme protein production, thus such kind of strategy was not applied in our study.

### Evaluation of high-heme-producing *E. coli *strain for expression of *Bs*Dyp

**Fig. 5:**
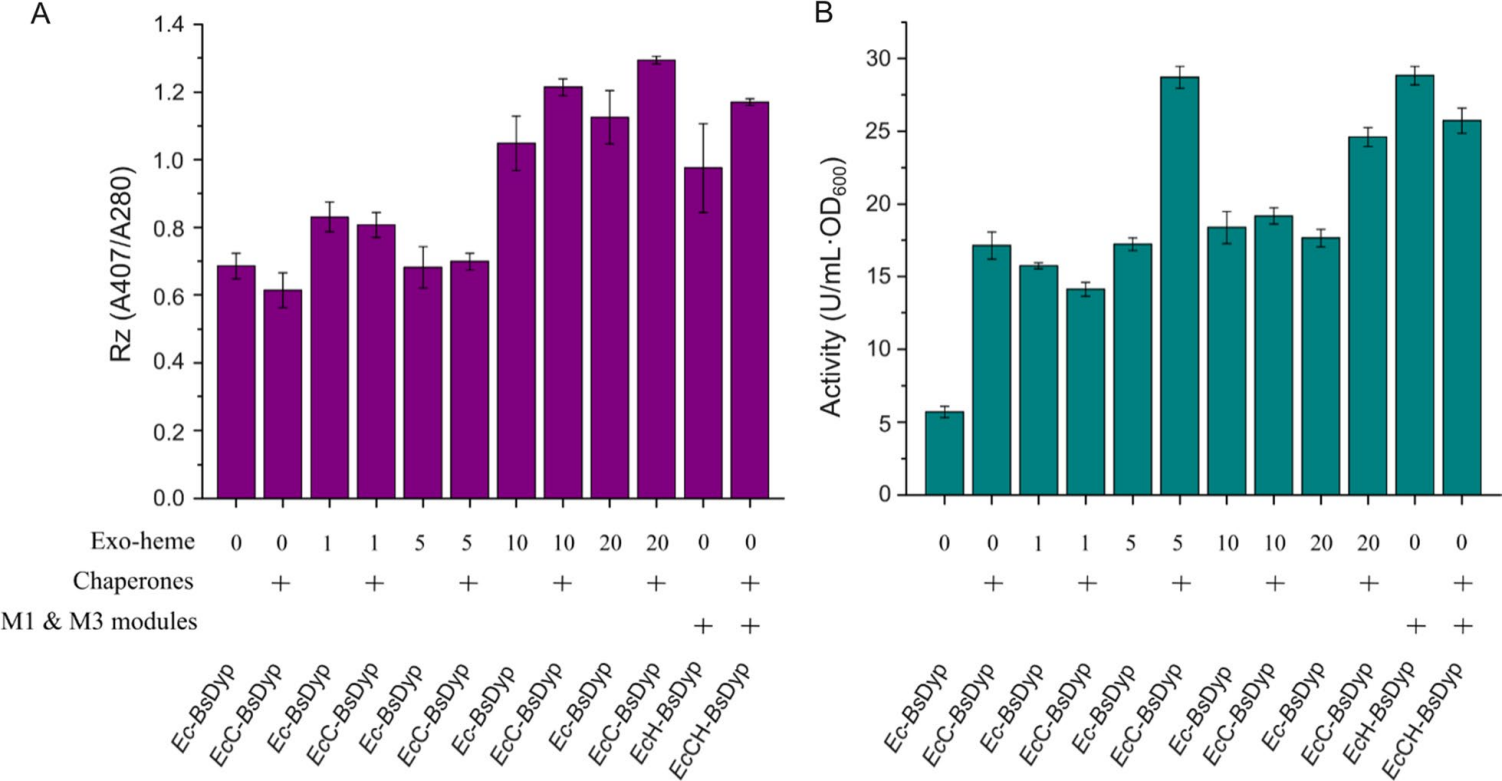
Characterization of *B. subtilis* dye-decolorizing peroxidase (*Bs*Dyp) purified from *E. coli* BL21(DE3) and *Ec*-M13 strains. **A** The Rz (A407/A280) ratio of purified *Bs*Dyp protein. **B** The enzyme activity of purified *Bs*Dyp protein. Exo-heme, exogenously supplemented with 0, 1, 5, 10 or 20 uM heme; Chaperones, co-expression of molecular chaperones including GroES, GroEL and Tig; M1 & M3 modules, overexpression of the 7 genes involved in module 1 and module 3 (*Ec*-M13 strain)

Dyp is another heme-containing peroxidase that can degrade various dyes, lignin-related compounds, and multiple mycotoxins and has drawn tremendous interest for biotechnological applications [25]. Therefore, the *Bs*Dyp was chosen as the first target protein to evaluate the potential of *Ec*-M13 strain, the high-heme-producing *E. coli* recombinant, for heme protein synthesis. The *Bs*Dyp overexpression plasmid was transformed into *Ec*-M13 and *E. coli* BL21(DE3) wild-type strains, creating *Ec*H_*Bs*Dyp and *Ec*_*Bs*Dyp mutants, respectively. The results showed that the Rz values (approximating the ratio of correctly assembled heme-bound protein to total protein) of *Bs*Dyp produced by *Ec*_*Bs*Dyp were increased as the concentration of exogenous heme increased (Fig. 5A), suggesting that the heme biosynthesis in *E. coli* wild-type strain is not sufficient for efficient production of heme proteins. With the addition of exogenous heme, the *Bs*Dyp activities of *Ec*_*Bs*Dyp were also significantly enhanced (Fig. 5B). When the concentration of exogenous heme was 10 μM, the Rz value and activity of *Bs*Dyp of *Ec*_*Bs*Dyp increased by 52.2% and 221.9%, respectively, compared to the controls. The intracellular heme production of *Ec*-M13 strain was about 2 μM/OD_600_ (the final OD_600_ of the fermentation culture was about 2). When *Bs*Dyp was overexpressed in *Ec*-M13, the Rz value of *Bs*Dyp of *Ec*H_*Bs*Dyp was comparable to that of *Ec*_*Bs*Dyp fermented with addition of 10 μM exogenous heme and the activity of *Bs*Dyp of *Ec*H_*Bs*Dyp was increased by 57.0%, compared to that of *Ec*_*Bs*Dyp fermented with addition of 10 μM exogenous heme, indicating that the intracellular heme production of *Ec*-M13 (approximately 4 μM) was sufficient for efficient *Bs*Dyp production.

It has been reported that molecular chaperone proteins could assist the correct folding of *Bs*Dyp [26]. Thus, the effect of co-expression of three molecular chaperones (GroES, GroEL and Tig) on *Bs*Dyp production was also investigated. The molecular chaperone overexpression plasmid pG-Tf2 was transformed into *Ec*_*Bs*Dyp and *Ec*H_*Bs*Dyp, generating *Ec*C_*Bs*Dyp and *Ec*CH_*Bs*Dyp strains, respectively. Compared to *Ec*_*Bs*Dyp, the Rz values of *Bs*Dyp of *Ec*C_*Bs*Dyp were slightly increased when high exogenous heme (10 and 20 μM) was used (Fig. 5A). Meanwhile, overexpression of molecular chaperones could also slightly increase the Rz value of *Bs*Dyp of *Ec*CH_*Bs*Dyp compared to that of *Ec*H_*Bs*Dyp. When molecular chaperones were overexpressed and 5 μM exogenous heme was supplemented, the *Bs*Dyp activity of *Ec*C_*Bs*Dyp reached 28.7 U/mL·OD_600_, which was enhanced by about fourfold compared to the control and comparable to that of *Ec*H_*Bs*Dyp (28.8 U/mL·OD_600_) without exogenous heme addition and molecular chaperone overexpression. Collectively, the high-heme-producing *Ec*-M13 strain could serve as a powerful chassis for the production of heme-containing peroxidases.

### Application of *Ec*-M13 strain for the production of hemoglobin, myoglobin and leghemoglobin

**Fig. 6:**
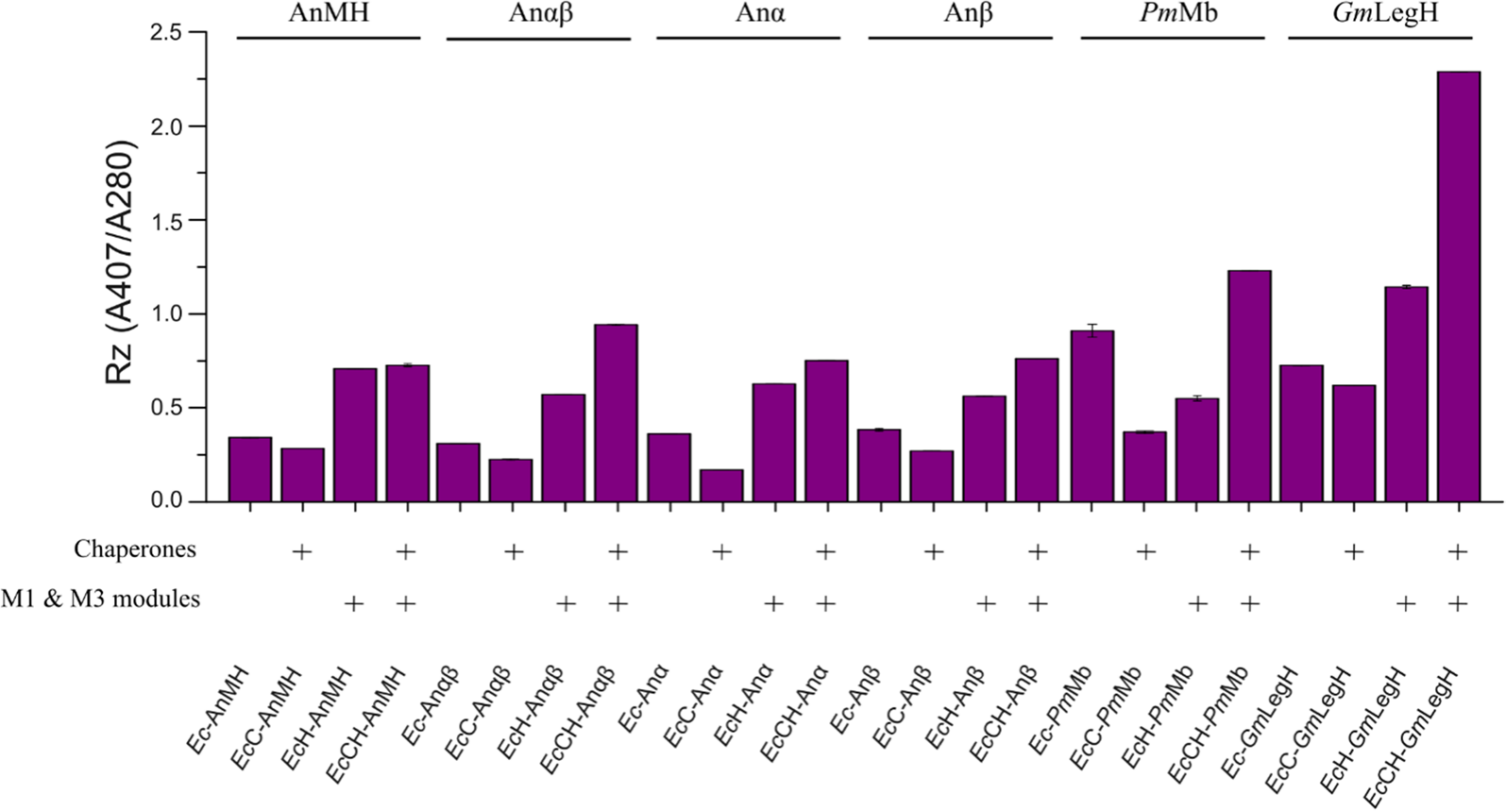
Characterization of hemoglobin (AnMH, Anαβ, Anα and Anβ), myoglobin (*Pm*Mb) and leghemoglobin (*Gm*LegH) purified from *E. coli* BL21(DE3) and *Ec*-M13 strains. AnMH, the common ancestor of myoglobin and hemoglobin; Anαβ, the common ancestor of globin α and β; Anα, the ancestor of globin α; Anβ, the ancestor of globin β; *Pm*Mb, myoglobin derived from *P. macrocephalus*; *Gm*LegH, leghemoglobin derived from *G. max*

Hemoglobin, myoglobin and leghemoglobin are well-known oxygen-binding proteins that are able to deliver oxygen in vertebrates and plants and could be used in clinical treatment and food industry. Therefore, the expressions of these three kinds of oxygen-transport proteins, including reconstructed ancestral vertebrate hemoglobin (AnMH, Anαβ, Anα and Anβ) [27], myoglobin (*Pm*Mb) from *Physeter macrocephalus*, and leghemoglobin (*Gm*LegH) from *Glycine max*, were also tested in *Ec*-M13 strain without or with co-expression of molecular chaperones (namely *Ec*H or *Ec*CH strain, respectively). The wild-type strain *E. coli* BL21(DE3) was used as the control strain. As a monomer, AnMH is the common ancestor of hemoglobin and myoglobin. The Rz value of AnMH expressed in *Ec*-M13 was increased by 107.0%, compared to that expressed in wild-type strain (Fig. 6). Co-expression of molecular chaperones didn’t increase the Rz value of monomeric AnMH. The Anαβ, Anα and Anβ evolved from AnMH and formed homodimers or homotetramers. Compared with the controls, the Rz values (A407/A280 ratio) of these hemoglobins were also significantly improved when expressed in *Ec*-M13 stain. It is noteworthy that co-expression of molecular chaperones in *Ec*-M13 could further increase the Rz values of these homodimers and homotetramers, indicating that molecular chaperones would facilitate the assembly of heme and globin proteins with complex structure. The Rz values of Anαβ, Anα and Anβ expressed in *Ec*CH were increased by 204.9%, 108.3% and 98.2%, respectively, compared to that expressed in wild-type strain (Fig. 6). The *Pm*Mb and *Gm*LegH are representative oxygen carriers in whales and legumes. Results showed that expression of *Pm*Mb in *Ec*-M13 didn’t enhance its Rz value compared to that expressed in wild-type strain, whereas the synergistic action of molecular chaperone expression and high intracellular heme (*Ec*CH strain) enhanced the Rz value of *Pm*Mb by 35.2% (Fig. 6). Similar to the results obtained with Anαβ, Anα and Anβ, using *Ec*-M13 as the expression host and further co-expression of molecular chaperones had positive effects on the assembly of heme and *Gm*LegH protein. The Rz value of *Gm*LegH of *Ec*CH_*Gm*LegH reached 2.3, which was enhanced by 2.2-fold compared to the control (Fig. 6). Together, these results demonstrate that *Ec*-M13 strain is a suitable host for efficient synthesis of hemoglobin, myoglobin and leghemoglobin.

### Application of *Ec*-M13 strain for cytochrome P450 expression and whole-cell bioconversion

**Fig. 7:**
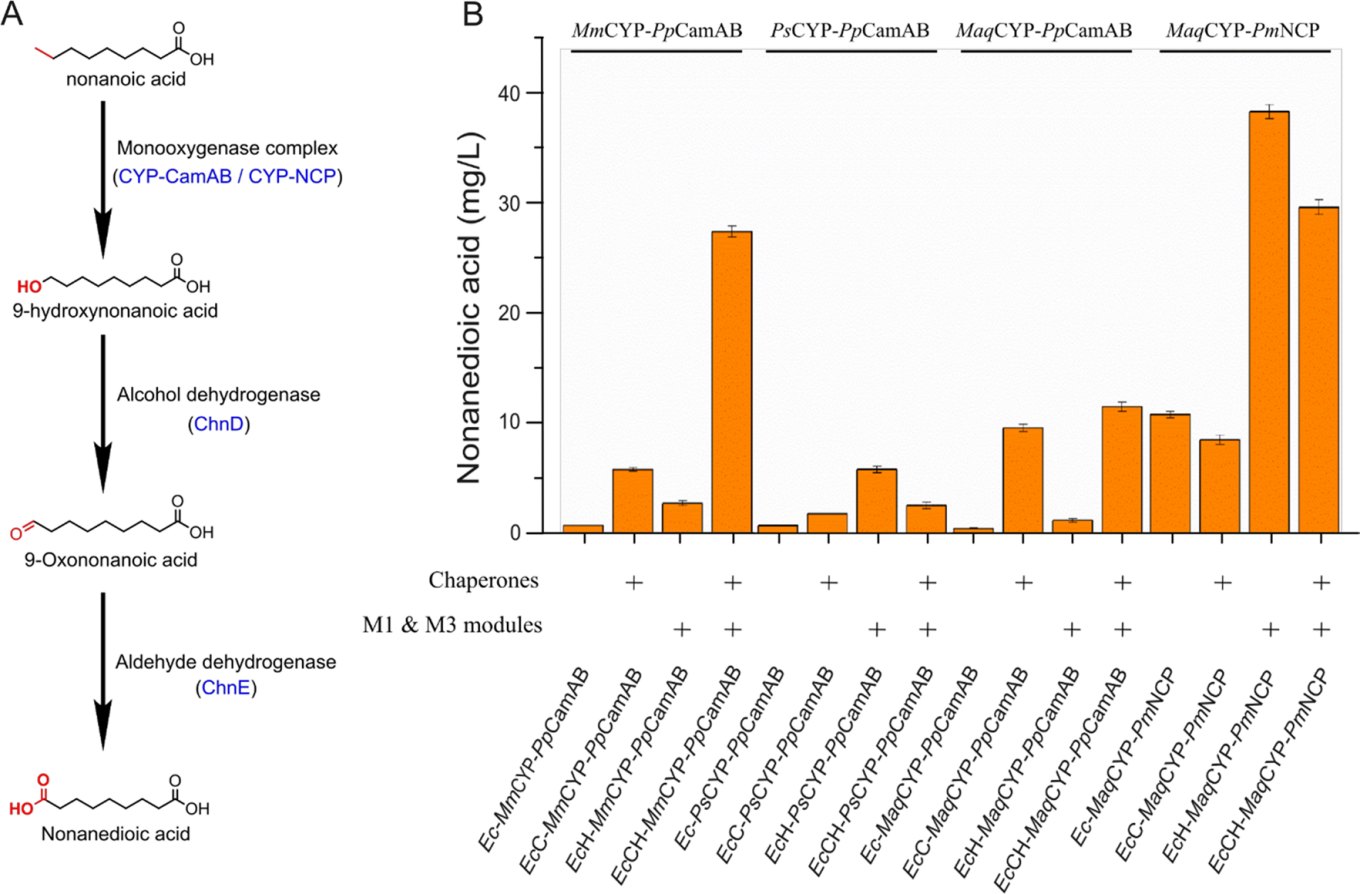
Application of *Ec*-M13 strain for whole-cell P450 bioconversion for nonanedioic acid production. **A** The synthetic pathway of nonanedioic acid from nonanoic acid. **B** Nonanedioic acid production. Three CYP153A subfamily cytochrome P450 (CYP) enzymes derived from *M. marinum* (*Mm*CYP), *Polaromonas* sp. JS666 (*Ps*CYP), and *M. aquaeolei* (*Maq*CYP) were tested. *P. putida* putidaredoxin reductase and putidaredoxin *(Pp*CamAB*)* and *P. megaterium* NADPH-cytochrome P450 oxidoreductase (*Pm*NCP) are NADH- and NADPH-dependent electron transport proteins, respectively, for reconstituting the activity of CYP enzymes

Cytochrome P450 enzymes (CYP) are a large family of heme-containing monooxygenases, capable of catalyzing a wide range of oxidative reactions. Due to their high degree of site-, regio- and stereo-selectivities, CYPs have been developed as versatile biocatalysts for the production of high-value biochemicals and drugs [28]. However, the poor stability and the inherent difficulty of heterologous expression limit their application. Here, the expressions of three CYP enzymes from *Mycobacterium marinum* (*Mm*CYP), *Polaromonas* sp. JS666 (*Ps*CYP), and *Marinobacter aquaeolei (Maq*CYP*)*, which belong to CYP153A subfamily and could catalyze the hydroxylation of alkanes and fatty acid terminals for fine and bulk chemicals synthesis [29], were tested in *Ec*-M13 strain for whole-cell bioconversion of nonanoic acid to nonanedioic acid (Fig. 7A). For reconstituting the activity of CYP enzymes, NADH-dependent electron transport proteins *Pseudomonas putida* putidaredoxin reductase (*Pp*CamA) and putidaredoxin (*Pp*CamB) were co-expressed along with molecular chaperones. Compared with the controls, expression of CYP enzymes in *Ec*-M13 strain could significantly increase the nonanedioic acid production (Fig. 7B). When *Ps*CYP was used, the nonanedioic acid production of *Ec*H_*Ps*CYP_*Pp*CamAB was enhanced by 6.5-fold compared to that of *Ec*_*Ps*CYP_*Pp*CamAB, indicating that the high-heme-producing strain could significantly promote the functional expression of CYP enzymes. In addition, co-expression of molecular chaperones showed positive effect on nonanedioic acid productions of *Ec*CH_*Mm*CYP_*Pp*CamAB and *Ec*CH_*Maq*CYP_*Pp*CamAB, which were increased by 8.3- and 8.8-fold, respectively, compared to those of *Ec*H_*Mm*CYP_*Pp*CamAB and *Ec*H_*Maq*CYP_*Pp*CamAB, respectively (Fig. 7B). Furthermore, another NADPH-cytochrome P450 oxidoreductase from *Priestia megaterium* (*Pm*NCP) was used to replace *Pp*CamAB and tested in *Maq*CYP overexpression hosts. Results showed that co-expression of *Maq*CYP and *Pm*NCP in *Ec*-M13 strain could greatly improve the nonanedioic acid production (Fig. 7B). The final titer of nonanedioic acid of *Ec*H_*Maq*CYP_*Pm*NCP reached 38.6 mg/L, which was increased by 33.8-fold compared to that of *Ec*H_*Maq*CYP_*Pp*CamAB, indicating that NADPH-dependent electron transport protein was more suitable for reconstitution of the activity of *Maq*CYP enzyme. To sum up, the high-heme-producing *Ec*-M13 strain could be useful for the development of whole-cell biocatalysts involving CYP enzymes for high-value biochemicals and drugs production.

## Conclusions

In this study, *K. phaffii* and *E. coli*, two widely used recombinant protein expression platforms, were selected as the hosts to construct heme-producing microbial chassis for high-value heme proteins production. Functional expression of heme protein was failed in engineered *K. phaffii* strain due to the accumulation of intermediates of heme synthesis. High intracellular heme production (as high as 1.04 mg/L·OD_600_) was successfully achieved in engineered *E. coli* strain Ec-M13 after systematic optimization of the native C5 pathway-based heme synthetic route. Finally, functional expression of Dyp, hemoglobin, myoglobin, leghemoglobin and CYP enzymes in Ec-M13 was confirmed. Enhanced assembly efficiencies and activities of these heme proteins were achieved. In summary, results from this work provide valuable references for constructing high-efficient cell factories for the production of heme and difficult-to-express heme proteins. The engineered *Ec*-M13 strain with sufficient supply of intracellular heme could serve as a valuable host for efficient synthesis of heme proteins with high yields and activities.

## Materials and methods

### Strains, media and chemicals

*K. phaffii* GS115 was initially used as the parental strain for engineering and heme production. YPD medium, BMGY medium and BMMY medium were employed for cultivation and selection of *K. phaffii* strains [30]. *E. coli* strains DH5α and BL21(DE3) were used as the hosts for plasmid amplification and target genes expression, respectively. LB medium (10 g/L tryptone, 5 g/L yeast extract, 10 g/L NaCl; 20 g/L agar for plates) was used for routine cloning works for *E. coli* strains. For promoting heme synthesis and heterologous heme proteins expression in shake-flask fermentation, recombinant *E. coli* strains were cultured in LBFG medium (10 g/L tryptone, 5 g/L yeast extract, 10 g/L NaCl, 30 mg/L FeSO_4_·7H_2_O, 20 g/L monosodium glutamate, 100 mM potassium phosphate, pH 6.0). 2,2-azino-bis (3-ethylbenzothiazoline-6sulfonic acid) (ABTS), 1,9-nonanedioic acid, 5-ALA were purchased from Sigma-Aldrich (St. Louis, MO, USA). Heme was purchased from TCI (Tokyo, Japan). Nonanoic acid sodium salt was purchased from Yuanye (Shanghai, China).

### Construction of plasmids and strains

To construct the heme-producing *K. phaffii* strain, the 8 genes involved in heme synthesis in *K. phaffii* were amplified, fused with strong constitutive promoter and further integrated into the genome of *K. phaffii* GS115 by homologous recombination using two plasmids (Additional file 1: Fig. S7). All primers used in this study are listed in Additional file 1: Table S3. In brief, the genes *hem1*, *hem2*, *hem3* and *hem4* were cloned into vector pPICZαA derivative, generating plasmid pPICZH1-4 (Additional file 1: Fig. S7). This plasmid was linearized by *Avr*II and transformed into *K. phaffii* GS115 via electroporation. The positive clones were screened by YPD plates containing 100 μg/mL zeocin. In order to isolate multiplex genome integration strains, the obtained clones were further screened by YPD plates containing gradually increased zeocin concentrations (200, 500, 1,000 and 2,000 μg/mL). Five colonies resistant to 2000 μg/mL zeocin were obtained and one of them was designated as *Pp*H4. Subsequently, plasmid pPICNH12-15 was constructed by inserting the *hem12*, *hem13*, *hem14* and *hem15* genes into pPICZαA derivative (Additional file 1: Fig. S7). Then, pPICNH12-15 was digested by *Av*rII and transformed into *K. phaffii Pp*H4 strain. Using the similar screening method, the positive clones were screened on YPD plates containing 100, 200, 500, 1,000 and 2,000 μg/mL nourseothricin. Finally, one strain which could be resistant to 2,000 μg/mL nourseothricin was designated as *Pp*H8.

To construct the heme-producing *E. coli* strain, the genes encoding glutamyl-tRNA synthetase (GltX), glutamyl-tRNA reductase (HemA), glutamate-1-semialdehyde-2,1-aminomutase (HemL), HemB, HemC, HemD, HemE, HemF, HemG and HemH were amplified from the genomic DNA of *E. coli* BL21(DE3). The amplicons were digested with appropriate restriction enzymes and ligated with pCDFDuet-1, pETDuet-1 or pRSFDuet-1 vectors [18]. The plasmids harboring different combinations of heme synthesis related genes were listed in Additional file 1: Table S1. Plasmids were introduced into *E. coli* BL21(DE3) for heme production. Transformants were selected on LB plates containing streptomycin (100 μg/mL), ampicillin (100 μg/mL) or kanamycin (100 μg/mL). All strains were listed in Additional file 1: Table S2. The genes encoding heme proteins including horseradish peroxidase (HRP),* B. subtilis* dye-decolorizing peroxidase (*Bs*Dyp), reconstructed ancestral vertebrate hemoglobin (AnMH, Anαβ, Anα and Anβ) [27], *P. macrocephalus* myoglobin (*Pm*Mb), *G. max* leghemoglobin (*Gm*LegH) and three CYP enzymes from *M. marinum* (*Mm*CYP), *P.* sp. JS666 (*Ps*CYP) and *M. aquaeolei* (*Maq*CYP) [31] were codon-optimized and synthesized by GenScript (Nanjing, China). *P. putida* putidaredoxin reductase (*Pp*CamA) and putidaredoxin (*Pp*CamB) [29], *P. megaterium* NADPH-cytochrome P450 oxidoreductase (*Pm*NCP) [32], *Acinetobacter* sp. strain SE19 alcohol dehydrogenase (*As*ChnD) and aldehyde dehydrogenase (*As*ChnE) [33] were synthesized. The heme protein genes were ligated with pPIC9k, pColdI or pETDuet-1 vector. Plasmid pG-Tf2 harboring three molecular chaperones (GroES, GroEL and Tig) was used to study globin folding [26]. The expression of *groES*, *groEL* and *tig* genes were induced by tetracycline (5 ng/mL). All plasmids and strains used for heme proteins expression are listed in Additional file 1: Table S1 and Table S2, respectively.

### Cultivation of recombinant strains

For testing the heme and heme proteins productions in *K. phaffii* recombinants, the single colonies were inoculated into YPD medium and grown at 30 °C in a shaking incubator (200 rpm) for 48 h. The obtained cultures were transferred to BMGY medium at 1% inoculum and continued to be cultured for 48 h. Then, the cells were harvested by centrifugation at 4500 rpm for 5 min and resuspended in BMMY medium. After another 48 h of fermentation, the supernatant and cell lysis were used to investigate the heme and heme proteins productions. In order to explore the influence of precursors and carbon sources on heme and heme proteins productions, different concentrations of 5-ALA, glycine, iron ions, glucose, glycerol or methanol were added.

For testing the heme and heme proteins productions in *E. coli* recombinants, transformants harboring target genes were grown in LB medium containing appropriate antibiotics. Overnight cultures were then transferred to fresh LBFG medium (2% inoculum) containing appropriate antibiotics and cultured at 30 °C, 200 rpm. When the OD_600_ reached about 0.4–0.5, 0.5 mM IPTG and/or 5 ng/mL tetracycline was supplemented as needed and the cultures were fermented for another 48 h at 30 °C, 200 rpm (for heme production) or another 20 h at 16 °C, 200 rpm (for heme proteins production).

### Purification and characterization of heme proteins

The induced cells were harvested by centrifugation and further resuspended in binding buffer (pH 7.4, 20 mM Tris/HCl, 500 mM NaCl). The heme proteins were released by sonication at 130 W for 30 min and then purified using immobilized metal affinity chromatography with washing buffer (pH 7.4 20 mM Tris/HCl, 500 mM NaCl, 40 mM imidazole) and elution buffer (pH 7.4 20 mM Tris/HCl, 500 mM NaCl, 200 mM imidazole). The purified heme proteins were analyzed by SDS-PAGE. The protein concentration was determined by a Protein Assay kit (TIANGEN, Beijing, China). The UV–visible spectroscopy analysis of heme protein was performed in the range of 230 to 800 nm in the 20 mM pH 5.0 malonate buffer by a microplate detector (BioTek Synergy H1, VT, USA). Typical heme-binding enzymes have a Soret band at 403–408 nm [34]. Heme proteins investigated in this study including hemoglobin, myoglobin, peroxidase also exhibited characteristic spectra, indicating that heme was successfully incorporated into the target proteins forming the mature heme proteins. The Rz (A407/A280) ratio of purified target proteins was used to characterize the purity of the heme-bound protein [35]. The activities of HRP and *Bs*Dyp were determined using the method described by Qin et al. [26].

### Whole-cell bioconversion of nonanoic acid to nonanedioic acid

The induced cells were harvested by centrifugation, washed with sterile ddH_2_O, and finally resuspended in 15 mL reaction solution (100 mM potassium phosphate buffer, pH 6.5, 2 g/L sodium nonanoate, 20 g/L glucose and 10 ml/L trace metal solution) at OD_600_ ≈ 5.0. Ingredients of trace metal solution were: 10 g/L FeSO_4_, 2.25 g/L ZnSO_4_, 1.0 g/L CuSO_4_, 0.5 g/L MnSO_4_, 0.23 g/L Na_2_B_4_O_7_, 2.0 g/L CaCl_2_, and 0.1 g/L (NH_4_)_6_Mo_7_O_24_. The bioconversion was performed in a shaking incubator (250 rpm) at 30 °C. The products generated in the reaction system were extracted by an equal volume of ethyl acetate and then the organic phase was collected by centrifugation. The products were redissolved in acetone after drying by a rotary vacuum dryer and derivatized using an equal volume of BSTFA (N,O-bis(trimethylsilyl)trifluoroacetamide) for 10 min. The alkylated products were analyzed after diluting with hexane.

### Analytical methods

Detection of 5-ALA was carried out following the protocol as described previously [36]. The reaction system, containing a mixture of 300 μL fermentation broth and 400 μL sodium acetate buffer (82.0 g anhydrous sodium acetate and 57.0 mL glacial acetic acid dissolved in 1 L H_2_O), was heated in a boiling water bath for 15 min after adding 35 μL acetylacetone. Upon cooling, 700 μL of modified Ehrlich’s agent was supplemented. The final reaction system was measured at a wavelength of 556 nm. The amount of heme was measured using reversed-phase HPLC (Shimadzu, Kyoto, Japan) with a UV-Vis detector (SPD-20A). Samples were injected and separated in the ZORBAX Eclipse Plus C18 column (Agilent, CA, USA) using methanol (buffer A) and 0.1% trifluoroacetic water (buffer B) as mobile phase. During this process, 30% methanol was used for 1 min with a flow rate of 1 mL/min and then the methanol concentration was gradually increased to 70% within 14 min and maintained for 15 min. The heme concentration was determined at 400 nm. To analyze the heme synthesis intermediates, LC-MS (Agilent 1100 series liquid chromatograph coupled to an Agilent LC/MSD VL mass spectrometer) equipped with Eclipse XDB-C18 column was used. The mobile phase was methanol (buffer A) and 0.1% formic acid water (buffer B). Samples were scanned in positive ion mode (ESI +) in the range of m/z = 200 to 900. The capillary voltage was set at 2.5 kV. Nitrogen was used as the drying gas at a flow rate of 12 L/min at 350 °C. The nebulizer pressure was set at 30 psig.

Samples from nonanoic acid bioconversion experiments were analyzed by Agilent 7890A gas chromatography equipped with an Agilent HP-5 ms capillary column and nitrogen was used as the carrier gas (flow rate, 1 mL/min). The injection volume was 1 μL and the sample was injected in a split-free mode. The column oven was set at 80 °C for 1 min, raised to 280 °C at a rate of 15 °C/min, held isotherm for 2 min. The samples were further analyzed by a GC-MS system (Q-Exactive GC Orbitrap, Thermo Scientific, MA, USA) equipped with a Thermo Scientific TraceGOLD TG-5SilMS column and helium was used as the carrier gas (flow rate, 1.2 mL/min). Mass spectra were collected using electrospray ionization. The injection volume was 1 μL and the sample was injected in a split ratio of 20:1. The column oven was set at 80 °C for 2 min, raised to 300 °C at a rate of 20 °C/min, held isotherm for 6 min. The samples were scanned in positive ion mode (ESI +) in the range of m/z = 30 to 550.

## Supplementary Information

**Additional file 1: Table S1.** Plasmids used in this study. **Table S2.** Strains used in this study. **Table S3.** Primers used in this study. **Figure S1.** The UV-visible spectroscopy analysis of heme standard (heme std) and fermentation products of GS115 and *Pp*H8 from 320 to 600 nm. **Figure S2.** UV-visible spectroscopy analysis (at 400 nm) of fermentation product of *Pp*H8 cultured in the medium with addition of different precursors. A 5-ALA. B Fe^2+^. C Glycine. The fermentation without addition of precursors was carried out as control (ck). **Figure S3.** UV-visible spectroscopy analysis (at 400 nm) of fermentation product of *Pp*H8 cultured in the medium with addition of different carbon sources. A Glucose. B Glycerol. C Methanol. The fermentation carried out in BMMY medium was used as control (ck). **Figure S4.** HPLC analysis of fermentation extracts of *E. coli* recombinants overexpressing different heme synthesis pathway genes. X, *gltX*; A, *hemA*; L, *hemL*; B, *hemB*; C, *hemC*; D, *hemD*; E, *hemE*; F, *hemF*; G, *hemG*; H, *hemH*. The heme and intermediates were marked with red and blue arrows, respectively. Three independent HPLC profiles were provided for each recombinant. **Figure S5.** HPLC analysis of fermentation extracts of *E. coli* recombinants co-expressing M1 and different genes in M2. M1 contains *gltX*, *hemA* and *hemL *genes; B, *hemB*; C, *hemC*; D, *hemD*; The heme and intermediates were marked with red and blue arrows, respectively. Five independent HPLC profiles were provided for each recombinant. **Figure S6.** Heme and intermediates productions and growth curves of strain *Ec*-M123 harboring plasmids pCDF-XAL, pET-BCD and pRSF-EFGH cultured in the LBFG medium supplemented with different concentrations of streptomycin (Str), kanamycin (Kan) and ampicillin (Amp). A HPLC profiles. B Growth curves. C heme productions. The concentrations of three antibiotics are indicated in μg/mL. **Figure S7.** The maps of recombinant plasmids pPICZH1-4 and pPICZH12-15 harboring heme synthesis pathway genes of *K. phaffii*. A Plasmid pPICZH1-4 containing *hem1*, *hem2*, *hem3* and *hem4* genes from *K. phaffii*. B Plasmid pPICZH12-15 containing *hem12*, *hem13*, *hem14* and *hem15* genes from *K. phaffii*.

## Data Availability

All data generated or analyzed during this study are included in this article and its additional information file.
