# Supplementary material for: Engineering Escherichia coli for efficient assembly of heme proteins
Source: Microb Cell Fact. 2023 Mar 28;22:59. doi: 10.1186/s12934-023-02067-5 (PMC10053478; doi:10.1186/s12934-023-02067-5)
Supplement: Supplementary file 1 — Additional file 1: Table S1. Plasmids used in this study. Table S2. Strains used in this study. Table S3. Primers used in this study. Figure S1. The UV-visible spectroscopy analysis of heme standard (heme std) and fermentation products of GS115 and PpH8 from 320 to 600 nm. Figure S2. UV-visible spectroscopy analysis (at 400 nm) of fermentation product of PpH8 cultured in the medium with addition of different precursors. A 5-ALA. B Fe2+. C Glycine. The fermentation without addition of precursors was carried out as control (ck). Figure S3. UV-visible spectroscopy analysis (at 400 nm) of fermentation product of PpH8 cultured in the medium with addition of different carbon sources. A Glucose. B Glycerol. C Methanol. The fermentation carried out in BMMY medium was used as control (ck). Figure S4. HPLC analysis of fermentation extracts of E. coli recombinants overexpressing different heme synthesis pathway genes. X, gltX; A, hemA; L, hemL; B, hemB; C, hemC; D, hemD; E, hemE; F, hemF; G, hemG; H, hemH. The heme and intermediates were marked with red and blue arrows, respectively. Three independent HPLC profiles were provided for each recombinant. Figure S5. HPLC analysis of fermentation extracts of E. coli recombinants co-expressing M1 and different genes in M2. M1 contains gltX, hemA and hemL genes; B, hemB; C, hemC; D, hemD; The heme and intermediates were marked with red and blue arrows, respectively. Five independent HPLC profiles were provided for each recombinant. Figure S6. Heme and intermediates productions and growth curves of strain Ec-M123 harboring plasmids pCDF-XAL, pET-BCD and pRSF-EFGH cultured in the LBFG medium supplemented with different concentrations of streptomycin (Str), kanamycin (Kan) and ampicillin (Amp). A HPLC profiles. B Growth curves. C heme productions. The concentrations of three antibiotics are indicated in μg/mL. Figure S7. The maps of recombinant plasmids pPICZH1-4 and pPICZH12-15 harboring heme synthesis pathway genes of K. phaffii. A Pla [file 12934_2023_2067_MOESM1_ESM.docx]

*Additional data*

# Engineering *Escherichia coli* for efficient assembly of heme proteins

Jianzhong Ge, Xiaolu Wang, Yingguo Bai, Yaru Wang, Yuan Wang, Tao Tu, Xing Qin, Xiaoyun Su, Huiying Luo, Bin Yao, Huoqing Huang*, Jie Zhang*

State Key Laboratory of Animal Nutrition, Institute of Animal Science, Chinese Academy of Agricultural Sciences, Beijing 100193, China

* Corresponding author:

Huoqing Huang

No.2 Yuanmingyuan West Road, Haidian district

Beijing, 100193 China

Tel: +86-10-62599910

E-mail: huanghuoqing@caas.cn

Jie Zhang

No.2 Yuanmingyuan West Road, Haidian district

Beijing, 100193 China

Tel: +86-10-62599910

E-mail: zhangjie09@caas.cn

**Table S1** Plasmids used in this study

| **Plasmid** | **Characteristics** | **Sources** |
| --- | --- | --- |
|  | **The plasmids expressed in *K. phaffii* strain** |  |
| pPICZα | *K. phaffii* expression plasmid, Zeo^r^ | Laboratory |
| pPIC9K | *K. phaffii* expression plasmid, G418^r^ | Laboratory |
| pPICZH1-4 | pPICZαA derivative for *hem1*, *hem2*, *hem3* and *hem4* overexpression, Zeo^r^ | This study |
| pPICNH12-15 | pPICZαA derivative for *hem12*, *hem13*, *hem14* and h*em15* overexpression, Ntc^r^ | This study |
| pPIC9K-HRP | horseradish peroxidase gene integrated into pPIC9K | Laboratory |
|  | **The plasmids expressed in *E. coli* strain** |  |
| pCDFDute-1 | Expression vector, CloDF13 ori, double T7lac promoters, Str^r^ | Novagen |
| pETDute-1 | Expression vector, ColE1 ori, double T7lac promoters, Amp^r^ | Novagen |
| pRSFDute-1 | Expression vector, RSF1030 ori, double T7lac promoters, Kan^r^ | Novagen |
| pColdI | Expression vector, ColE1 ori, cspA promoters Amp^r^ | Novagen |
| pG-Tf2 | Expression vector, p15A ori, pzt-1 promoters, encoding three molecular chaperones: groES, groEL and tig, Cam^r^ | Laboratory |
| pCDF-X | *gltX* from *E. coli* integrated into pCDFDute-1 | This study |
| pCDF-A | *hemA* from *E. coli* integrated into pCDFDute-1 | This study |
| pCDF-L | *hemL* from *E. coli* integrated into pCDFDute-1 | This study |
| pCDF-XA | *hemA* from *E. coli* integrated into pCDF-X | This study |
| pCDF-XL | *hemL* from *E. coli* integrated into pCDF-X | This study |
| pCDF-AL | *hemL* from *E. coli* integrated into pCDF-A | This study |
| pCDF-XAL | *hemL* from *E. coli* integrated into pCDF-XA | This study |
| pET-B | *hemB* from *E. coli* integrated into pETDute-1 | This study |
| pET-C | *hemC* from *E. coli* integrated into pETDute-1 | This study |
| pET-D | *hemD* from *E. coli* integrated into pETDute-1 | This study |
| pET-BC | *hemC* from *E. coli* integrated into pET-B | This study |
| pET-BD | *hemD* from *E. coli* integrated into pET-B | This study |
| pET-CD | *hemD* from *E. coli* integrated into pET-C | This study |
| pET-BCD | *hemD* from *E. coli* integrated into pET-BC | This study |
| pRSF-E | *hemE* from *E. coli* integrated into pRSFDute-1 | This study |
| pRSF-F | *hemF* from *E. coli* integrated into pRSFDute-1 | This study |
| pRSF-G | *hemG* from *E. coli* integrated into pRSFDute-1 | This study |
| pRSF-H | *hemH* from *E. coli* integrated into pRSFDute-1 | This study |
| pRSF-EF | *hemF* from *E. coli* integrated into pRSF-E | This study |
| pRSF-EG | *hemG* from *E. coli* integrated into pRSF-E | This study |
| pRSF-EH | *hemH* from *E. coli* integrated into pRSF-E | This study |
| pRSF-FG | *hemG* from *E. coli* integrated into pRSF-F | This study |
| pRSF-FH | *hemH* from *E. coli* integrated into pRSF-F | This study |
| pRSF-GH | *hemH* from *E. coli* integrated into pRSF-G | This study |
| pRSF-EFG | *hemG* from *E. coli* integrated into pRSF-EF | This study |
| pRSF-EFH | *hemH* from *E. coli* integrated into pRSF-EF | This study |
| pRSF-EGH | *hemE* from *E. coli* integrated into pRSF-GH | This study |
| pRSF-FGH | *hemF* from *E. coli* integrated into pRSF-GH | This study |
| pRSF-EFGH | *hemH* from *E. coli* integrated into pRSF-EFG | This study |
| pCold-AnMH | AnMH gene integrated into pColdI | This study |
| pCold-Anαβ | Anαβ gene integrated into pColdI | This study |
| pCold-Anα | Anα gene integrated into pColdI | This study |
| pCold-Anβ | Anβ gene integrated into pColdI | This study |
| pCold-*Pm*Mb | *Pm*Mb gene integrated into pColdI | This study |
| pCold-*Gm*LegH | *Gm*LegH gene integrated into pColdI | This study |
| pCold-*Bs*Dyp | Dyp enzymes gene from *B. subtilis* SCK6 integrated into pETDute-1 | This study |
| pET-*As*ChnD | *Acinetobacter* sp. strain SE19 alcohol dehydrogenase gene (*As*ChnD), codon optimized, synthesized and integrated into pETDute-1 | This study |
| pET-*As*ChnDE | *Acinetobacter* sp. strain SE19 aldehyde dehydrogenase gene (*As*ChnE), codon optimized, synthesized and integrated into pET-*As*ChnD | This study |
| pET-*As*ChnDE-*Pp*CamAB | *Pseudomonas putida* putidaredoxin reductase (CamA) and putidaredoxin (CamB) genes synthesized and integrated into pET-*As*ChnDE | This study |
| pET-*As*ChnDE-*Pp*CamAB-*Mm*CYP | CYP153 enzyme gene from *Mycobacterium marinum* codon optimized, synthesized and integrated into pET-*As*ChnDE-*Pp*CamAB | This study |
| pET-*As*ChnDE-*Pp*CamAB-*Maq*CYP | CYP153 enzyme gene from *Marinobacter aquaeolei* codon optimized, synthesized and integrated into pET-*As*ChnDE-*Pp*CamAB | This study |
| pET-*As*ChnDE-*Pp*CamAB-*Ps*CYP | CYP153 enzyme gene from *Polaromonas sp.* strain JS666 codon optimized, synthesized and integrated into pET-*As*ChnDE-*Pp*CamAB | This study |
| pET-*As*ChnDE-*Maq*CYP_*Pm*NCP | CYP153 enzyme gene from *Marinobacter aquaeolei* and NADPH-cytochrome P450 oxidoreductase (NCP) gene from *Priestia megaterium* codon optimized, synthesized and integrated into pET-*As*ChnDE | This study |

**Table S2** Strains used in this study

| **Strain** | **Characteristics** | **Sources** |
| --- | --- | --- |
|  | ***K. phaffii* strains for target gene expression** |  |
| GS115 | Wild-type *K. phaffii* strain | Laboratory |
| *pP*H4 | GS115 with linearized pPICZH1-4 integrated into *FLD1up* locus; Zeo^r^ | This study |
| *pP*H8 | GS115 with linearized pPICNH12-15 integrated into *FLD1up* locus; Zeo^r^ and Ntc^r^ | This study |
| *pP*H8-HRP | *pP*H8 with linearized pPIC9K-HRP integrated into *AOX1* locus; G418^r^ | This study |
| GS115-HRP | GS115 with linearized pPIC9K-HRP integrated into *AOX1* locus; G418^r^ | This study |
|  | ***E. coli* strains for target gene expression** |  |
| BL21(DE3) | F^-^ *omp*T *hsd*S(rB^-^, mB^-^) *gal dcm* (DE3) | Laboratory |
| *Ec*-X | BL21(DE3) harboring pCDF-X | This study |
| *Ec*-A | BL21(DE3) harboring pCDF-A | This study |
| *Ec*-L | BL21(DE3) harboring pCDF-L | This study |
| *Ec*-XA | BL21(DE3) harboring pCDF-XA | This study |
| *Ec*-XL | BL21(DE3) harboring pCDF-XL | This study |
| *Ec*-AL | BL21(DE3) harboring pCDF-AL | This study |
| *Ec*-M1 | BL21(DE3) harboring pCDF-XAL | This study |
| *Ec*-B | BL21(DE3) harboring pET-B | This study |
| *Ec*-C | BL21(DE3) harboring pET-C | This study |
| *Ec*-D | BL21(DE3) harboring pET-D | This study |
| *Ec*-BC | BL21(DE3) harboring pET-BC | This study |
| *Ec*-BD | BL21(DE3) harboring pET-BD | This study |
| *Ec*-CD | BL21(DE3) harboring pET-CD | This study |
| *Ec*-M2 | BL21(DE3) harboring pET-BCD | This study |
| *Ec*-E | BL21(DE3) harboring pRSF-E | This study |
| *Ec*-F | BL21(DE3) harboring pRSF-F | This study |
| *Ec*-G | BL21(DE3) harboring pRSF-G | This study |
| *Ec*-H | BL21(DE3) harboring pRSF-H | This study |
| *Ec*-EF | BL21(DE3) harboring pRSF-EF | This study |
| *Ec*-EG | BL21(DE3) harboring pRSF-EG | This study |
| *Ec*-EH | BL21(DE3) harboring pRSF-EH | This study |
| *Ec*-FG | BL21(DE3) harboring pRSF-FG | This study |
| *Ec*-FH | BL21(DE3) harboring pRSF-FH | This study |
| *Ec*-GH | BL21(DE3) harboring pRSF-GH | This study |
| *Ec*-EFG | BL21(DE3) harboring pRSF-EFG | This study |
| *Ec*-EFH | BL21(DE3) harboring pRSF-EFH | This study |
| *Ec*-EGH | BL21(DE3) harboring pRSF-EGH | This study |
| *Ec*-FGH | BL21(DE3) harboring pRSF-FGH | This study |
| *Ec*-M3 | BL21(DE3) harboring pRSF-EFGH | This study |
| *Ec*-M1-B | BL21(DE3) harboring pCDF-XAL and pET-B | This study |
| *Ec*-M1-C | BL21(DE3) harboring pCDF-XAL and pET-C | This study |
| *Ec*-M1-D | BL21(DE3) harboring pCDF-XAL and pET-D | This study |
| *Ec*-M1-BC | BL21(DE3) harboring pCDF-XAL and pET-BC | This study |
| *Ec*-M1-BD | BL21(DE3) harboring pCDF-XAL and pET-BD | This study |
| *Ec*-M1-CD | BL21(DE3) harboring pCDF-XAL and pET-CD | This study |
| *Ec*-M12 | BL21(DE3) harboring pCDF-XAL and pET-BCD | This study |
| *Ec*-M1-E | BL21(DE3) harboring pCDF-XAL and pRSF-E | This study |
| *Ec*-M1-F | BL21(DE3) harboring pCDF-XAL and pRSF-F | This study |
| *Ec*-M1-G | BL21(DE3) harboring pCDF-XAL and pRSF-G | This study |
| *Ec*-M1-H | BL21(DE3) harboring pCDF-XAL and pRSF-H | This study |
| *Ec*-M1-EF | BL21(DE3) harboring pCDF-XAL and pRSF-EF | This study |
| *Ec*-M1-EG | BL21(DE3) harboring pCDF-XAL and pRSF-EG | This study |
| *Ec*-M1-EH | BL21(DE3) harboring pCDF-XAL and pRSF-EH | This study |
| *Ec*-M1-FG | BL21(DE3) harboring pCDF-XAL and pRSF-FG | This study |
| *Ec*-M1-FH | BL21(DE3) harboring pCDF-XAL and pRSF-FH | This study |
| *Ec*-M1-GH | BL21(DE3) harboring pCDF-XAL and pRSF-GH | This study |
| *Ec*-M1-EFG | BL21(DE3) harboring pCDF-XAL and pRSF-EFG | This study |
| *Ec*-M1-EFH | BL21(DE3) harboring pCDF-XAL and pRSF-EFH | This study |
| *Ec*-M1-EGH | BL21(DE3) harboring pCDF-XAL and pRSF-EGH | This study |
| *Ec*-M1-FGH | BL21(DE3) harboring pCDF-XAL and pRSF-FGH | This study |
| *Ec*-M13 | BL21(DE3) harboring pCDF-XAL and pRSF-EFGH | This study |
| *Ec*-M123 | BL21(DE3) harboring pCDF-XAL, pET-BCD and pRSF-EFGH | This study |
| *Ec*-AnMH | BL21(DE3) harboring pCold-AnMH | This study |
| *Ec*C-AnMH | BL21(DE3) harboring pG-Tf2 and pCold-AnMH | This study |
| *Ec*H-AnMH | *Ec*-M13 harboring pCold-AnMH | This study |
| *Ec*CH-AnMH | *Ec*-M13 harboring pG-Tf2 and pCold-AnMH | This study |
| *Ec*-*Pm*Mb | BL21(DE3) harboring pCold-*Pm*Mb | This study |
| *Ec*C-*Pm*Mb | BL21(DE3) harboring pG-Tf2 and pCold-*Pm*Mb | This study |
| *Ec*H-*Pm*Mb | *Ec*-M13 harboring pCold-*Pm*Mb | This study |
| *Ec*CH-*Pm*Mb | *Ec*-M13 harboring pG-Tf2 and pCold-*Pm*Mb | This study |
| *Ec*-Anαβ | BL21(DE3) harboring pCold-Anαβ | This study |
| *Ec*C-Anαβ | BL21(DE3) harboring pG-Tf2 and pCold-Anαβ | This study |
| *Ec*H-Anαβ | *Ec*-M13 harboring pCold-Anαβ | This study |
| *Ec*CH-Anαβ | *Ec*-M13 harboring pG-Tf2 and pCold-Anαβ | This study |
| *Ec*-Anα | BL21(DE3) harboring pCold-Anα | This study |
| *Ec*C-Anα | BL21(DE3) harboring pG-Tf2 and pCold-Anα | This study |
| *Ec*H-Anα | *Ec*-M13 harboring pCold-Anα | This study |
| *Ec*CH-Anα | *Ec*-M13 harboring pG-Tf2 and pCold-Anα | This study |
| *Ec*-Anβ | BL21(DE3) harboring pCold-Anβ | This study |
| *Ec*C-Anβ | BL21(DE3) harboring pG-Tf2 and pCold-Anβ | This study |
| *Ec*H-Anβ | *Ec*-M13 harboring pCold-Anβ | This study |
| *Ec*CH-Anβ | *Ec*-M13 harboring pG-Tf2 and pCold-Anβ | This study |
| *Ec*-*Gm*LegH | BL21(DE3) harboring pCold-*Gm*LegH | This study |
| *Ec*C-*Gm*LegH | BL21(DE3) harboring pG-Tf2 and pCold-*Gm*LegH | This study |
| *Ec*H-*Gm*LegH | *Ec*-M13 harboring pCold-*Gm*LegH | This study |
| *Ec*CH-*Gm*LegH | *Ec*-M13 harboring pG-Tf2 and pCold-*Gm*LegH | This study |
| *Ec*-*Bs*Dyp | BL21(DE3) harboring pCold-*Bs*Dyp | This study |
| *Ec*C-*Bs*Dyp | BL21(DE3) harboring pG-Tf2 and pCold-*Bs*Dyp | This study |
| *Ec*H-*Bs*Dyp | *Ec*-M13 harboring pCold-*Bs*Dyp | This study |
| *Ec*CH-*Bs*Dyp | *Ec*-M13 harboring pG-Tf2 and pCold-*Bs*Dyp | This study |
| *Ec*-*Mm*CYP-*Pp*CamAB | BL21(DE3) harboring pET-*As*ChnDE-*Pp*CamAB-*Mm*CYP | This study |
| *Ec*C-*Mm*CYP-*Pp*CamAB | BL21(DE3) harboring pG-Tf2 and pET-*As*ChnDE-*Pp*CamAB-*Mm*CYP | This study |
| *Ec*H-*Mm*CYP-*Pp*CamAB | *Ec*-M13 harboring pET-*As*ChnDE-*Pp*CamAB-*Mm*CYP | This study |
| *Ec*CH-*Mm*CYP-*Pp*CamAB | *Ec*-M13 harboring pG-Tf2 and pET-*As*ChnDE-*Pp*CamAB-*Mm*CYP | This study |
| *Ec*-*Maq*CYP-*Pp*CamAB | BL21(DE3) harboring pET-*As*ChnDE-*Pp*CamAB-*Maq*CYP | This study |
| *Ec*C-*Maq*CYP-*Pp*CamAB | BL21(DE3) harboring pG-Tf2 and pET-*As*ChnDE-*Pp*CamAB-*Maq*CYP | This study |
| *Ec*H-*Maq*CYP-*Pp*CamAB | *Ec*-M13 harboring pET-*As*ChnDE-*Pp*CamAB-*Maq*CYP | This study |
| *Ec*CH-*Maq*CYP-*Pp*CamAB | *Ec*-M13 harboring pG-Tf2 and pET-*As*ChnDE-*Pp*CamAB-*Maq*CYP | This study |
| *Ec*-*Ps*CYP-*Pp*CamAB | BL21(DE3) harboring pET-*As*ChnDE-*Pp*CamAB-*Ps*CYP | This study |
| *Ec*C-*Ps*CYP-*Pp*CamAB | BL21(DE3) harboring pG-Tf2 and pET-*As*ChnDE-*Pp*CamAB-*Ps*CYP | This study |
| *Ec*H-*Ps*CYP-*Pp*CamAB | *Ec*-M13 harboring pET-*As*ChnDE-*Pp*CamAB-*Ps*CYP | This study |
| *Ec*CH-*Ps*CYP-*Pp*CamAB | *Ec*-M13 harboring pG-Tf2 and pET-*As*ChnDE-*Pp*CamAB-*Ps*CYP | This study |
| *Ec*-*Maq*CYP-*Pm*NCP | BL21(DE3) harboring pET-*As*ChnDE-*Maq*CYP_*Pm*NCP | This study |
| *Ec*C-*Maq*CYP- *Pm*NCP | BL21(DE3) harboring pG-Tf2 and pET-*As*ChnDE-*Maq*CYP_*Pm*NCP | This study |
| *Ec*H-*Maq*CYP-*Pm*NCP | *Ec*-M13 harboring pET-*As*ChnDE-*Maq*CYP_*Pm*NCP | This study |
| *Ec*CH-*Maq*CYP-*Pm*NCP | *Ec*-M13 harboring pG-Tf2 and pET-*As*ChnDE-*Maq*CYP_*Pm*NCP | This study |

**Table S3** Primers used in this study

| **Primer** | **sequence (5’**-**3’)** |
| --- | --- |
| **Used to construct recombinant plasmids expressed in *K. phaffii* strain** | |
| pPICZH1-4 construction | tgactagtccccacacaccatagcttca |
|  | ggcagatctgatctcatgaccaaaatcc |
|  | tcagatctgcccacacaccatagcttca |
|  | tttgtaattaaaacttagattagattgctatgc |
|  | atctaagttttaattacaaaatgccaaaagccattcttctg |
|  | aaaagaggcgacagttagtcgacccggacaacttttgcttcc |
|  | gactaactgtcgcctcttttatctgc |
|  | gagataacatgttggcgaataactaaaatgtatgtag |
|  | attcgccaacatgttatctccagtatcacaaaactacc |
|  | ggactagtcagaatcctggtccccaaaatag |
|  | gggaagcaaaagttgtccgggatccttttttgtagaaatgtcttgg |
|  | caaactccattgtgttttgatagttgttcaattgattg |
|  | atcaaaacacaatggagtttgtcgcccgt |
|  | gaggcgacagtggtcaacactggattaaggacatc |
|  | agtgttgaccactgtcgcctcttttatctgc |
|  | tatgcaccatgttggcgaataactaaaatgtatgtagtg |
|  | attcgccaacatggtgcataaggctgaatacttgg |
|  | gataaaagaggcgacagttatgaaccgtcgattgggctg |
| pPICNH12-15 construction | ctgtcgactccccacacaccatagcttcaaaatg |
|  | ccagatctgatctcatgaccaaaatcc |
|  | tcagatctggatccttttttgtagaaatgtcttgg |
|  | atctactcattgtgttttgatagttgttcaattgattg |
|  | cttcagttctggaaatctactcattgtgttttga |
|  | gtgggtctagactaacaccattgtcatccctg |
|  | tggtgttagtctagacccacacaccatagcttcaaaatg |
|  | cattttgtaattaaaacttagattagattgctatgc |
|  | taagttttaattacaaaatgcttaaccgtcgtttccaatc |
|  | gagtcgacagtcactccccctgaagaac |
|  | agggatgacaatggtgttagactgtcgcctcttttatctgc |
|  | cgatggccatgttggcgaataactaaaatgtatgtag |
|  | attcgccaacatggccatcgactctgatatc |
|  | cgacagttatgttgcaaatgtggtcaccagt |
|  | catttgcaacataactgtcgcctcttttatctgc |
|  | ttttcagcatgttggcgaataactaaaatgtatgtag |
|  | attcgccaacatgctgaaaagtcttgcacc |
|  | ttgaagctatggtgtgtgggtacctgatcatcatcaatctactgttc |
| **Used to construct recombinant plasmids expressed in *E. coli* strain** | |
| Construction of plasmids for overexpressing heme synthesis pathway module 1 | ggaattccatatgaaaatcaaaactcgcttc |
|  | ccgctcgagttactgctgattttcgcgt |
|  | catgccatgggtaccaagaagcttttagcactcggtatcaacc |
|  | cgcggatccctactccagcccgaggct |
|  | cgagctcataaaaggaggaaaatatatgagtaagtctgaaaatctttacag |
|  | aaggaaaaaagcggccgctcacaacttcgcaaacacc |
| Construction of plasmids for overexpressing heme synthesis pathway module 2 | catgccatgggtacagacttaatccaacgc |
|  | cgcggatccttaacgcagaatcttcttctcag |
|  | gggaattccatatgttagacaatgttttaagaattgc |
|  | gaagatcttcatgccggagcgtctc |
|  | gaagatctataaaaggaggaaaatatatgagtatcctggtcacccg |
|  | ccgctcgagttattgtaatgcccgtaaaagc |
| Construction of plasmids for overexpressing heme synthesis pathway module 3 | catgccatgggtaccgaacttaaaaacgatc |
|  | cgagctcttagcggtgatattgttcagac |
|  | cgagctcataaaaggaggaaaatatatgaaacccgacgcacac |
|  | aaaactgcagttacacccaatccctgacct |
|  | aaaactgcagataaaaggaggaaaatatgtgaaaacattaattcttttctcaac |
|  | aaggaaaaaagcggccgcttatttcagcgtcggtttgtc |
|  | ggaattccatatgcgtcagactaaaaccgg |
|  | ccgctcgagttagcgatacgcggcaac |


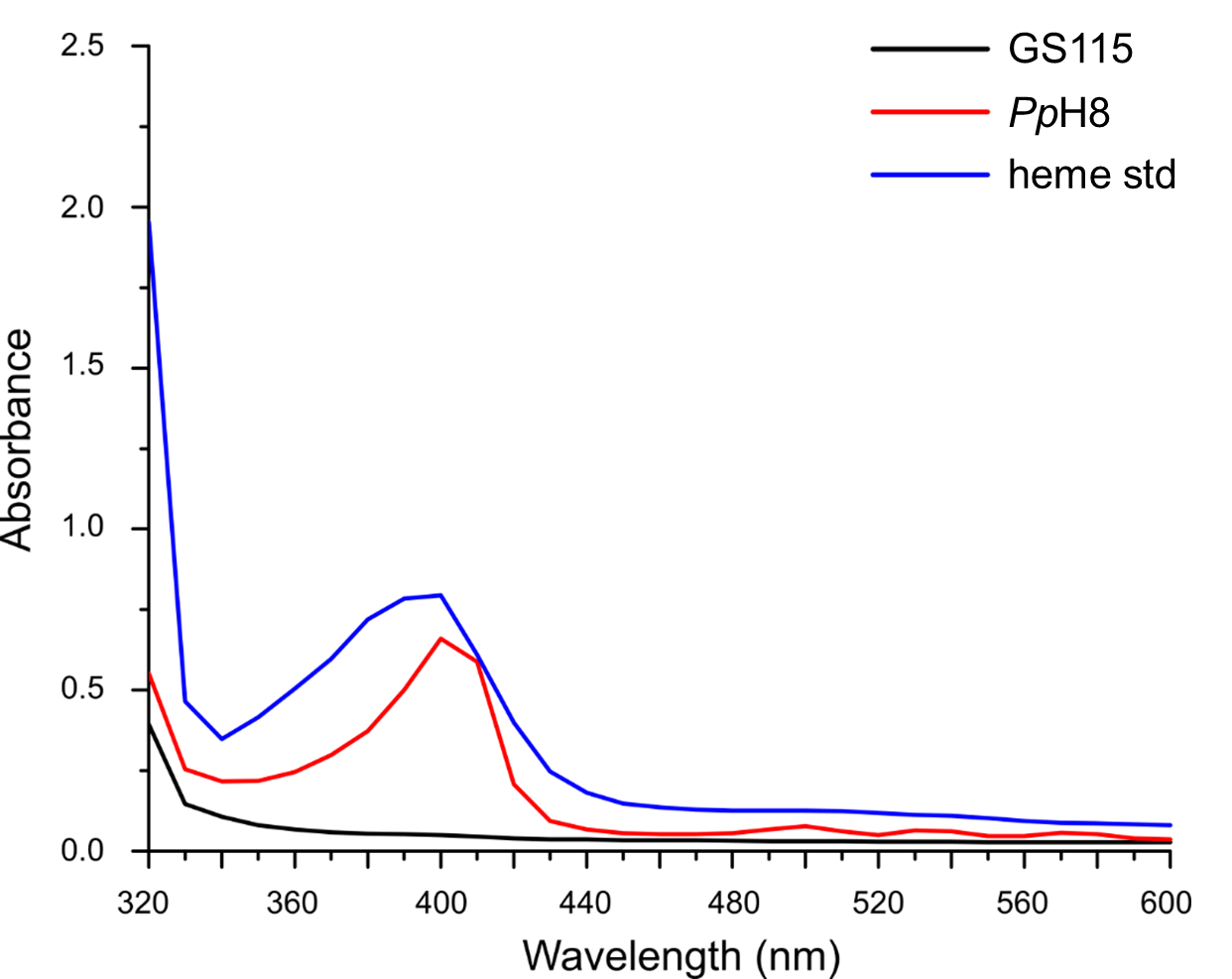


**Fig. S1** The UV-visible spectroscopy analysis of heme standard (heme std) and fermentation products of GS115 and *Pp*H8 from 320 to 600 nm.


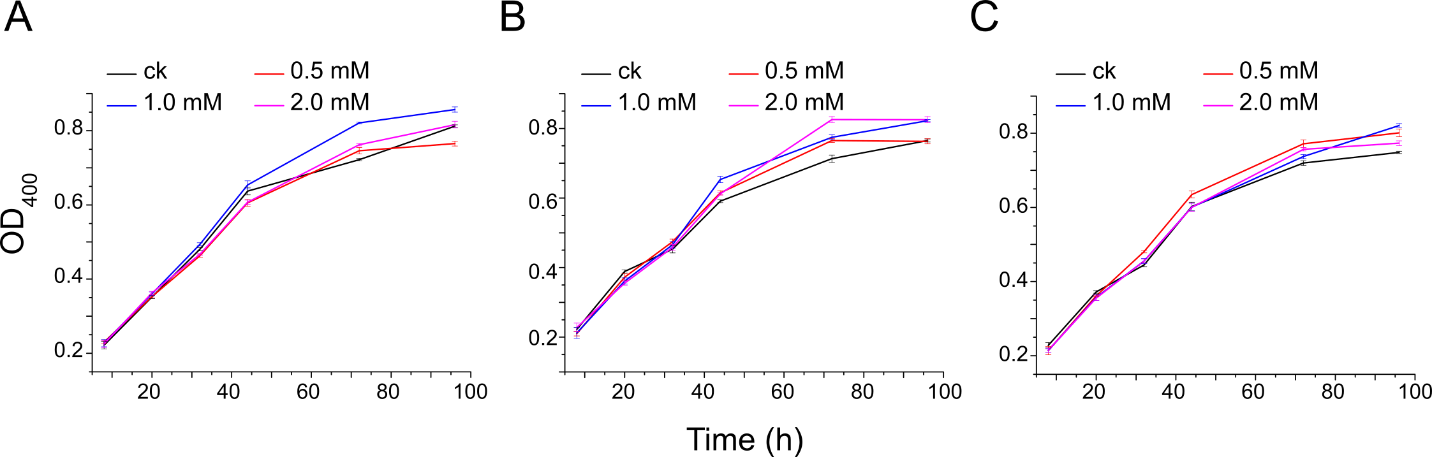


**Fig. S2** UV-visible spectroscopy analysis (at 400 nm) of fermentation product of *Pp*H8 cultured in the medium with addition of different precursors. **A** 5-ALA. **B** Fe^2+^. **C** Glycine. The fermentation without addition of precursors was carried out as control (ck).


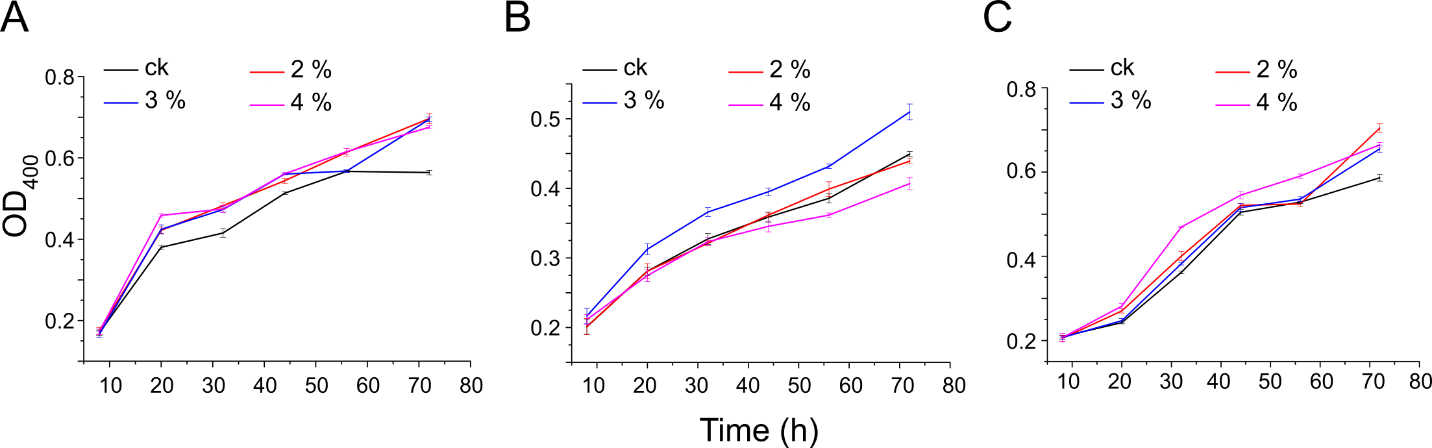


**Fig. S3** UV-visible spectroscopy analysis (at 400 nm) of fermentation product of *Pp*H8 cultured in the medium with addition of different carbon sources. **A** Glucose. **B** Glycerol. **C** Methanol. The fermentation carried out in BMMY medium was used as control (ck).


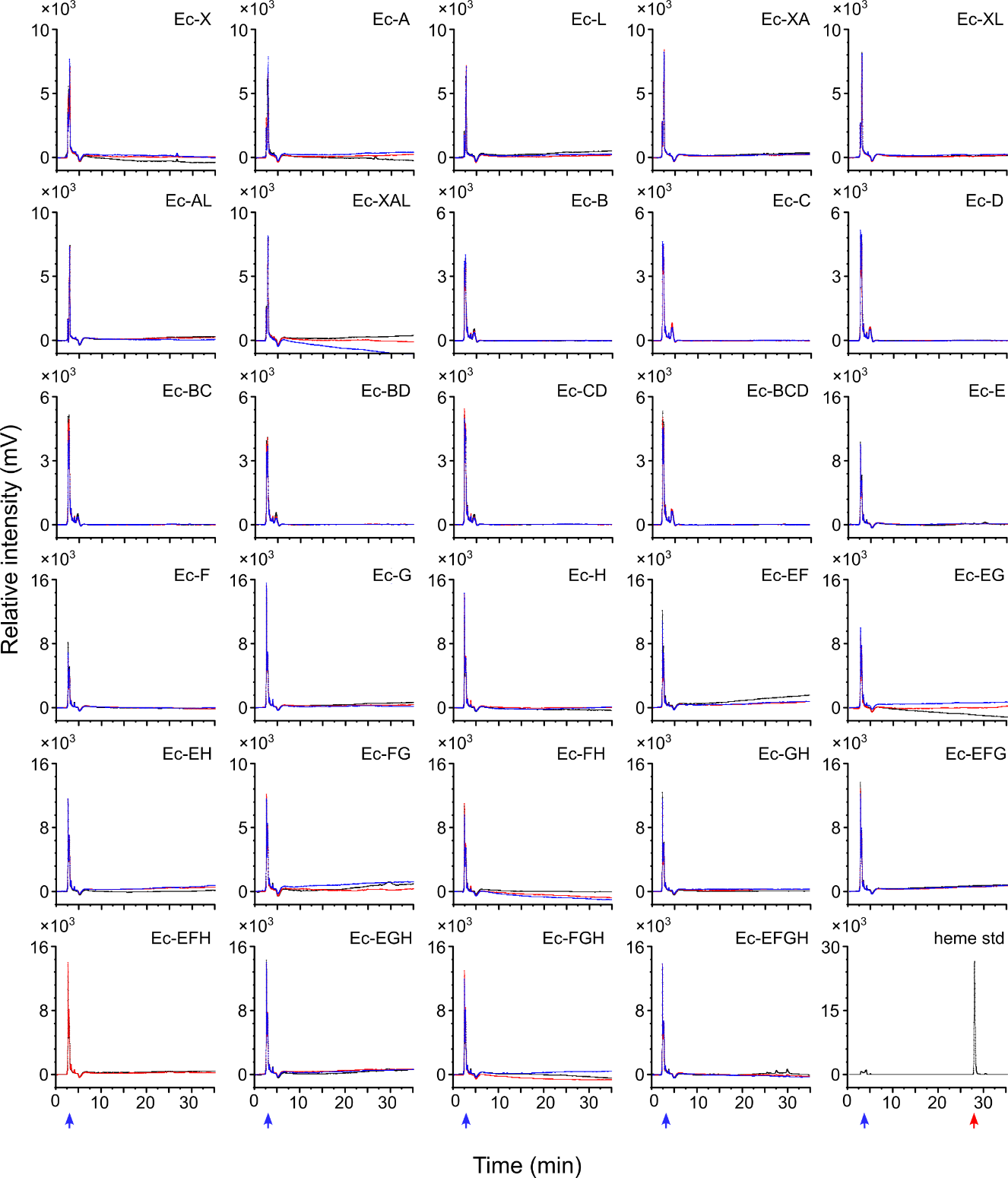


**Fig. S4** HPLC analysis of fermentation extracts of *E. coli* recombinants overexpressing different heme synthesis pathway genes. X, *gltX*; A, *hemA*; L, *hemL*; B, *hemB*; C, *hemC*; D, *hemD*; E, *hemE*; F, *hemF*; G, *hemG*; H, *hemH*. The heme and intermediates were marked with red and blue arrows, respectively. Three independent HPLC profiles were provided for each recombinant.


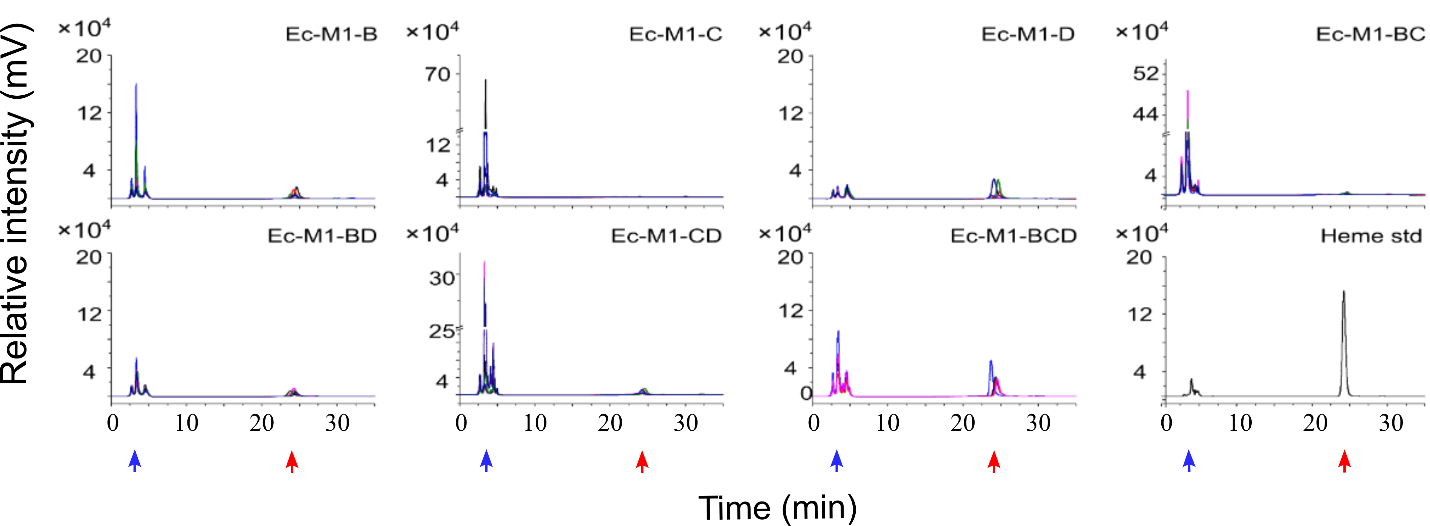


**Fig. S5** HPLC analysis of fermentation extracts of *E. coli* recombinants co-expressing M1 and different genes in M2. M1 contains *gltX*, *hemA* and *hemL* genes; B, *hemB*; C, *hemC*; D, *hemD*; The heme and intermediates were marked with red and blue arrows, respectively. Five independent HPLC profiles were provided for each recombinant.


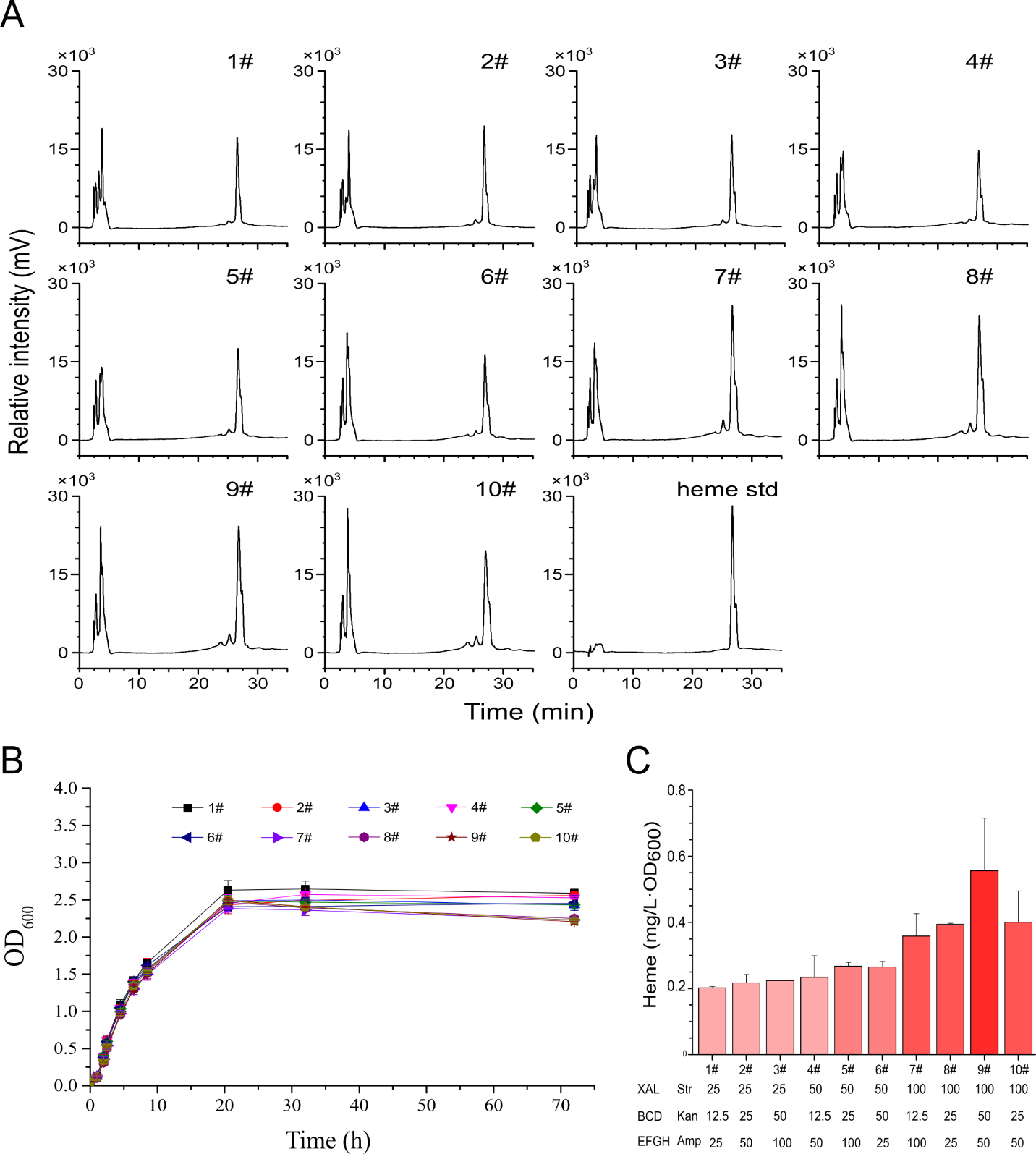


**Fig. S6** Heme and intermediates productions and growth curves of strain *Ec*-M123 harboring plasmids pCDF-XAL, pET-BCD and pRSF-EFGH cultured in the LBFG medium supplemented with different concentrations of streptomycin (Str), kanamycin (Kan) and ampicillin (Amp). **A** HPLC profiles. **B** Growth curves. **C** heme productions. The concentrations of three antibiotics are indicated in μg/mL.


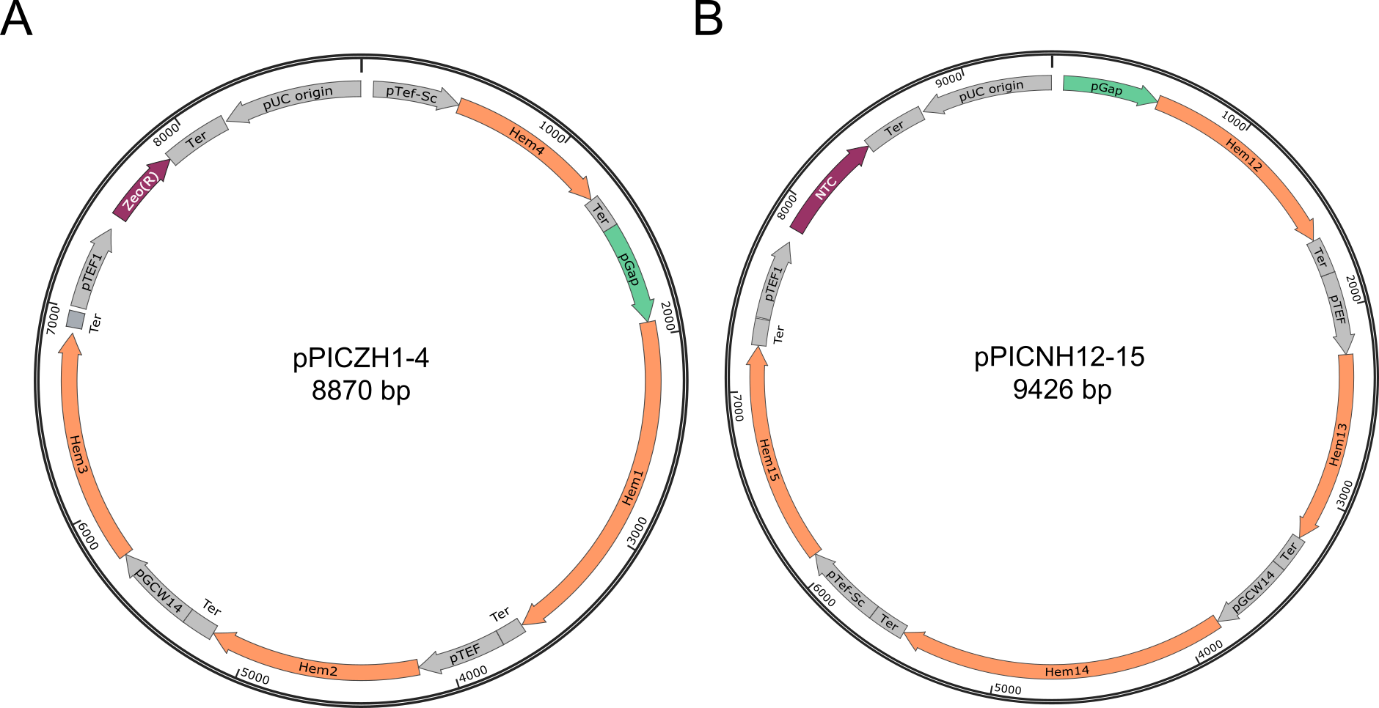


**Fig. S7** The maps of recombinant plasmids pPICZH1-4 and pPICZH12-15 harboring heme synthesis pathway genes of *K. phaffii*. **A** Plasmid pPICZH1-4 containing *hem1*, *hem2*, *hem3* and *hem4* genes from *K. phaffii*. **B** Plasmid pPICZH12-15 containing *hem12*, *hem13*, *hem14* and *hem15* genes from *K. phaffii*.
